# Psychological and neuro-morphological predictors of resilience in healthy adults: the whole is more than the sum of its parts

**DOI:** 10.3389/fnins.2025.1597835

**Published:** 2025-07-09

**Authors:** Carlo Fabrizio, Eleonora Picerni, Daniela Laricchiuta, Davide Decandia, Fabrizio Piras, Andrea Termine, Gianfranco Spalletta, Laura Petrosini, Debora Cutuli

**Affiliations:** ^1^IRCCS Santa Lucia Foundation, Rome, Italy; ^2^Department of Philosophy, Social Sciences & Education, University of Perugia, Perugia, Italy; ^3^Department of Psychology, Sapienza University, Rome, Italy

**Keywords:** psychological constructs, personality traits, brain volumes and thickness, Resilience Scale-10, Elastic Net

## Abstract

**Introduction:**

Research in resilience has shifted the focal point from a pathological orientation (responses to recovery from trauma or stress) to an emphasis on the role of resilience in health (protective factors to maintain health levels despite eventual adversities). Even if many single factors impact resilience capacities, an integrative predictive model including psychological constructs, personality traits, and brain structural features may offer a more profound knowledge of trait resilience.

**Methods:**

We examined the associations between Resilience Scale-10 scores with numerous psychological dimensions, personality traits, and neuro-morphological features (brain volumes and thickness) in 69 healthy adults of both sexes. Furthermore, we investigated the predictors potentially associated with resilience by regression modeling.

**Results:**

In the present exploratory study resilience values were predicted: positively by some personality characteristics (*Conscientiousness, Openness,* Resourcefulness, Enlightened second nature), psychological dimensions (Self-efficacy, Positive affect, Confidence), and brain morphological aspects (volumes of amygdala and hippocampus, and cortical thickness of temporal pole); and negatively by other personality traits (Fear of uncertainty) and psychological dimensions (Anxiety, Depression, Need for Approval).

**Discussion:**

The present results, although exploratory and hypothesis-generating rather than confirmatory, suggest that the identification of the multiple psychological and personality features and neuro-morphological aspects associated with resilience may represent a good step forward in understanding the factors that predispose individuals to be resilient.

## 1 Introduction

*Some tried to bury me, but they did not know I was a seed.*

*(Dinos Christianopoulos)*

*…but as they learn to see either the darkness alters or something in the sight adjusts itself to midnight and life steps almost straight.*

*(Emily Dickinson)*

Resilience can be considered a positive outcome despite adversity (Bonanno and Diminich, 2013; Kalisch et al., 2019; Park, 2023; Southwick and Charney, 2012). Resilient individuals face life adversities by implementing successful coping responses, enjoy intimate bonds and a wider social circle, express empathy to others, promote autonomous goals, live a creative and significant life, and are free of distressing symptoms in the face of trauma or grief (Feldman, 2020; Southwick and Charney, 2012; Tugade and Fredrickson, 2004). The majority of research on resilience has, up to now, focused on the outcomes of trauma, regarding resilience as the absence of symptoms or maintenance of homeostasis following trauma, limiting the scope of resilience to an observable phenomenon after an adverse event (Kalisch et al., 2015; Liu et al., 2023). Investigating the response to adversity as a “proxy” of resilience, many studies have examined the associations between psychological constructs and resilience in response to stressful events. These studies indicated that multiple factors, such as personality traits, self-efficacy, flexibility, optimism, and positive affect, may promote adaptive responses to adverse situations (Graham et al., 2021; Kalisch et al., 2015; Oshio et al., 2018). Similarly, many neuroimaging studies have reported resilience-related differences in response to different kinds of trauma in the volumes (Bolsinger et al., 2018; Bromis et al., 2018) and resting-state activity (Disner et al., 2018; Harnett et al., 2021; Liu et al., 2018; Long et al., 2019) of many brain areas.

Besides the conceptualization of resilience as an outcome, resilience has been also conceived as a process or a trait (Luthar et al., 2000; Fletcher and Sarkar, 2013; Kalisch et al., 2017). The outcome models assess resilience retrospectively, typically as positive functioning following adversity, while process models emphasize the dynamic, ongoing nature of resilience over time. In contrast, trait-based models view resilience as a relatively stable disposition or capacity that facilitates flexible adaptation to environmental demands, predisposing individuals to adapt to change (Troy et al., 2023; Waugh et al., 2008). While process and outcome perspectives have gained prominence, the trait conceptualization remains foundational and provides a valuable framework for identifying individual predisposition before an adversity occurs. Resilience can thus be approached by considering the biological and psychological underpinnings of such a construct *per se*, and not only as a reaction in the face of trauma or stress (Feldman, 2020; Kalisch et al., 2019; Lü et al., 2014). According to this approach, indices of resilience may include both the absence of ill-being and the presence of well-being and psychological health. Thus, one can conceptualize resilience as categorical (e.g., resilient versus not resilient) or continuous (e.g., the more resilient you are, the better able you’ll be to handle stress) (Troy et al., 2023). This shift in operationalizing resilience has led to greater interest in resilience-predisposing factors, in an attempt to gain insights into aspects of resilience not captured by models focused solely on responses following adversity (Windle, 2011). For example, the relationships between resilience and positive affect, emotion regulation, or personality traits have shown that individuals who effectively regulate their emotions, experience positive affect, or possess constructive personality traits tend to display greater resilience (Alessandri et al., 2014; Nieto et al., 2023; Oshio et al., 2018). Furthermore, other studies have demonstrated that resilience properties are determined by the adaptive responses of brain networks involved in emotional regulation, coping, and cognitive flexibility (Gupta et al., 2017; Kahl et al., 2020; Long et al., 2019; Palamarchuk and Vaillancourt, 2021; Shi et al., 2019; Shikimoto et al., 2021; van der Werff et al., 2013; Wu et al., 2013).

Our study adopts the trait perspective not to exclude the dynamic nature of resilience, but to better examine the ensemble of stable psychological and neurobiological factors that may underlie resilience capacity across the lifespan. This aligns with recent integrative approaches regarding resilience as a cohesive whole (Kalisch et al., 2019; Troy et al., 2023). Despite growing recognition that resilience involves complex interactions among many psychological traits and neurobiological mechanisms, most existing studies have examined these factors in isolation or within clinical populations. Few have employed integrative, multivariate approaches to model these interactions in healthy adults. Moreover, Machine Learning techniques such as Elastic Net regression remain underutilized in resilience research, despite their potential to identify predictive patterns from high-dimensional data.

The present study addresses this gap by applying Elastic Net regression to a multimodal dataset in a healthy adult sample to identify combined psychological and neurobiological predictors of trait resilience. In doing so, we aim to contribute a more holistic, data-driven understanding of resilience.

## 2 Methods

### 2.1 Participants

A sample of 69 healthy right-handed subjects (28 males: mean age ±SD: 38.21 ± 11.87 years (y), 41 females: 41.88 ± 12.69 y) reporting no history of psychiatric or neurological diseases participated in this study. Educational level ranged from an eighth grade to a post-graduate degree (mean education years ± SD: 15.75 ± 2.99 y). The present investigation was part of a larger project exploring the relations between brain and psychological dimensions (Picerni et al., 2019, 2021, 2022). Only those subjects who agreed to come again to the Santa Lucia Foundation to be tested on RS-10 and other psychological scales were enrolled in the current study. Data were filtered to keep only the participants who could contribute data for all variables included in the final analyses. All participants underwent MRI scanning and completed questionnaires. Inclusion and exclusion criteria are described in detail in the Supplementary materials.

### 2.2 Psychological assessment

Participants’ psychological profile was assessed through the Italian versions of the following self-report psychological questionnaires and scales: Resilience Scale-10 (RS-10), Attachment Style Questionnaire (ASQ), Beck’s Depression Inventory Scale (BDI), Coping Orientation to Problems and Experiences (COPE), Emotion Regulation Questionnaire (ERQ), General Self-Efficacy Scale (GSES), Hamilton Anxiety Rating Scale (HAM-A), Hamilton Depression Rating Scale (HAM-D), Holmes and Rahe Stressful Event Scale (HR-SS), Interpersonal Reactivity Index (IRI), Positive and Negative Affect Schedule (PANAS), State–Trait Anger Expression Inventory (STAXI), State–Trait Anxiety Inventory-Form Y (STAI-Y), Toronto Alexithymia Scale (TAS-20), Raven’s Progressive Matrices (RPM).

Participants’ personality traits were assessed through the Big Five Questionnaire-2 (BFQ-2) and Temperament and Character Inventory (TCI).

Italian versions of psychological and personality scales were used. Supplementary materials describe tests and questionnaires in detail and report descriptive statistics for socio-demographic and psychological variables (Supplementary Table S1).

#### 2.2.1 The Resilience Scale (RS-10)

Resilience was measured using the Italian version of RS-10 (Gerino et al., 2017; Peveri, 2010; Sagone and Caroli, 2014), a 10-item version of the psychometrically sound Resilience Scale (RS) (Wagnild and Young, 1993). Previous studies (Peveri, 2010; Rebagliati et al., 2016) demonstrated the equivalence between the unifactorial 10-item version (RS-10) and the original version of RS (encompassing 25 items and measuring five essential characteristics of resilience). The unifactorial structure of RS-10 facilitated the definition of the resilience measure, which was our dependent variable. The 10 items of the test are rated on a 7-point Likert scale. The RS-10 gives total scores ranging from 10 to 70, with higher scores reflecting greater levels of resilience.

### 2.3 MRI acquisition and processing

Participants underwent a neuroimaging protocol including standard clinical sequences (FLAIR, DP-T2-weighted) and a volumetric whole-brain 3D high-resolution T1-weighted sequence, performed with a 3 T Allegra MRI. Volumetric whole-brain T1-weighted images were obtained in the sagittal plane using a Modified Driven Equilibrium Fourier Transform (MDEFT) sequence (Echo Time/Repetition Time-TE/TR- = 2.4/7.92 ms, flip angle 15, voxel size 1 × 1 × 1 mm^3^). All planar sequence acquisitions were obtained in the plane of the AC-PC line. The FreeSurfer imaging analysis suite^1^ was used for reconstructing volumes and cortical thickness of brain regions (Dale et al., 1999; Fischl and Dale, 2000). Cerebellar parcellation was performed through a freely available patch-based multi-atlas segmentation tool called CERES (CEREbellum Segmentation) able to parcellate the cerebellar lobules automatically. CERES (Romero et al., 2017) is part of a broader software pipeline for volumetric brain analysis, namely volBrain.^2^

Neuroimaging data acquisition and processing are further detailed in the Supplementary materials.

### 2.4 Statistical analyses: data preparation and regression analysis

Before regression modeling, a Spearman correlation filter (Hollander et al., 2013) was used to select independent variables significantly correlated to the dependent variable RS-10 (Supplementary Table S3). The 279 variables taken into account for correlations were: socio-demographic variables (*n* = 2); psychological (*n* = 34) and personality (*n* = 37) variables from the 16 tests detailed in Supplementary Table S3; cortical volume variables (*n* = 66); sub-cortical volume variables (*n* = 26); cortical thickness variables (*n* = 66), cerebellar volume and thickness variables (*n* = 48). Sex was used as a between-subjects factor in non-parametric statistical analyses. Selected predictors, along with socio-demographics as covariates (namely, age, education, and sex), were used in a regression analysis to predict the resilience score. For the purposes of this exploratory investigation, we considered correlations with *p* ≤ 0.05 statistically significant. Then, to manage the presence of several predictors and to obtain further statistical reliability, the regression analysis was performed with the Elastic Net method. The Elastic Net modulates regression coefficients to penalize complex models implementing implicit feature selection through a regularization approach that combines ridge and LASSO regression (Kuhn and Johnson, 2013; Zou and Hastie, 2005). The model was trained using repeated k-fold cross-validation, with 5 folds repeated 5 times, to avoid overfitting and ensure the reliability of the results. Repeated k-fold cross-validation was preferred to single-run k-fold cross-validation because it ensures a more accurate estimate of results by reporting the mean result across all folds from all runs. Repeated cross-validation reduces the error in the estimate of mean model performance (Kohavi, 1995). On each run of this cross-validation procedure, data were centered and scaled. Further data transformations were avoided in order to ensure the regression results were directly interpretable. The model was evaluated using *R*^2^ and Root Mean Squared Error (RMSE) metrics. Feature importance was investigated by evaluating the model’s regression coefficients. All statistical analyses were performed in R.

## 3 Results

The frequency distribution of RS-10 scores is summarized in Supplementary Figure S1. No effect of the sex was found on RS-10 scores (Mann–Whitney U Test: *U* = 478.5, *p* = 0.245; RS-10 mean ± SD score: Males: 58.8 ± 7.4; Females: 55.2 ± 11.3). RS-10 was not significantly associated with age or education (Age *p* = 0.65; Education *p* = 0.98).

Furthermore, male and female participants did not differ in age (Mann–Whitney U Test: *U* = 453, *p* = 0.141), education level (Mann–Whitney U Test: *U* = 565, *p* = 0.916), or numerosity (Chi-Square = 2.45, *p* = 0.118).

Correlation tests were performed between RS-10 scores and all other psychological, personality, and neuroanatomical variables taken into account in this study. Out of these 279 variables examined, 46 exhibited a statistically significant correlation (*p*-value <0.05), as summarized in Table 1 (see also Supplementary Table S2. The complete list of the 279 correlations is reported in Supplementary Table S3). Namely, the first 21 variables of Table 1, which significantly correlated with RS-10 scores, concerned the psychological or personality domains. In particular, GSES (self-efficacy) and TCI-HA1 (Anticipatory worry of the subscale Harm Avoidance of TCI) displayed the strongest positive and negative correlations, respectively. The strongest correlations between RS-10 scores and brain morphological features concerned the cerebellar lobule X, the orbitofrontal cortex (OFC), and the amygdala. As expected, the psychological and personality variables related to confidence, openness, emotional stability, and positive emotions as well as the neuroanatomical variables related to the volume and thickness of cortical (e.g., OFC, temporal pole) and subcortical (e.g., amygdala, hippocampus) structures showed positive correlations (Table 1, variables colored in red). Accordingly, the psychological and personality variables related to distress, avoidance, and negative emotions as well as the neuroanatomical variables related to the volume and thickness of cerebellar lobules (X, I-II, VIIb) showed negative correlations (Table 1, variables colored in blue). By applying the False Discovery Rate (FDR) correction, 25 out of these 46 correlations were deemed significant (p-adjusted < 0.05). Given that our focus was to select variables with a numerical association with the resilience measure, and we were not interested in identifying potential false positive correlations when selecting the features for the Elastic Net, the 46 variables were selected as candidate predictors for the regression analysis, regardless of the p-value obtained through the application of the FDR correction.

**Table 1:** Significant correlations between the dependent variable RS-10 and the 46 candidate predictors for regression analysis (Elastic Net model).

Variables	Category	Spearman’s rho	*p*-value	FDR adjusted *p*-value
GSES	Psychological dimension	0.6507	**<0.00001**	**<0.00001**
PANAS - Positive Affect	Psychological dimension	0.6384	**<0.00001**	**<0.00001**
STAI-Y - Trait anxiety	Psychological dimension	−0.6050	**<0.00001**	**<0.00001**
IRI - Personal Distress	Psychological dimension	−0.5485	**<0.00001**	**0.0001**
STAI-Y - State Anxiety	Psychological dimension	−0.5249	**<0.00001**	**0.0002**
ASQ - Need for Approval	Psychological dimension	−0.5244	**<0.00001**	**0.0002**
ASQ -Confidence	Psychological dimension	0.4969	**0.00001**	**0.0006**
TCI - HA1	Personality trait	−0.4844	**0.00002**	**0.0008**
BFQ-2 Energy	Personality trait	0.4821	**0.00003**	**0.0008**
TCI - HA	Personality trait	−0.4804	**0.00003**	**0.0008**
TCI - SD3	Personality trait	0.4767	**0.00003**	**0.0009**
PANAS - Negative Affect	Psychological dimension	−0.4368	**0.0002**	**0.0041**
ASQ - Preoccupation with Relationships	Psychological dimension	−0.4301	**0.0002**	**0.0048**
BFQ-2 Emotional Stability	Personality trait	0.4123	**0.0004**	**0.0086**
HAM-D	Psychological dimension	−0.3991	**0.0007**	**0.0127**
STAXI - State Anger	Psychological dimension	−0.3952	**0.0008**	**0.0135**
ASQ - Relationships as Secondary	Psychological dimension	−0.3756	**0.0015**	**0.0242**
COPE - Problem solving	Psychological dimension	0.3714	**0.0017**	**0.0260**
TCI - SD2	Personality trait	0.3630	**0.0022**	**0.0320**
BFQ-2 Openness	Personality trait	0.3569	**0.0026**	**0.0364**
TCI - HA2	Personality trait	−0.3546	**0.0028**	**0.0372**
Left cerebellar lobule X	Cerebellar volume	−0.3513	**0.0031**	**0.0388**
BFQ-2 Conscientiousness	Personality trait	0.3500	**0.0032**	**0.0388**
TCI - SD	Personality trait	0.3467	**0.0035**	**0.0409**
Left medial orbitofrontal Cx	Cortical volume	0.3425	**0.0040**	**0.0443**
COPE Avoidance	Psychological dimension	−0.3358	**0.0048**	0.0515
BFQ-2 Lie	Personality trait	0.3311	**0.0054**	0.0563
Left amygdala	Subcortical volume	0.3246	**0.0065**	0.0648
BFQ-2 Agreeableness	Personality trait	0.3212	**0.0071**	0.0686
BDI - Total score	Psychological dimension	−0.3191	**0.0075**	0.0700
Left temporal pole	Cortical thickness	0.3155	**0.0083**	0.0745
Right cerebellar lobules I-II	Cortical thickness	−0.3037	**0.0112**	0.0976
Right hippocampus	Subcortical volume	0.2964	**0.0134**	0.1132
TCI - NS3	Personality trait	0.2831	**0.0184**	0.1510
TCI - HA4	Personality trait	−0.2659	**0.0272**	0.2170
ERQ Expressive Suppression	Psychological dimension	0.2606	**0.0305**	0.2345
Right pars orbitalis	Cortical volume	0.2591	**0.0316**	0.2345
Right cerebellar lobule X	Cortical thickness	−0.2586	**0.0319**	0.2345
Corpus callosum - central	Subcortical volume	0.2544	**0.0349**	0.2497
HAM-A	Psychological dimension	−0.2510	**0.0375**	0.2523
TAS-20 Total score	Psychological dimension	−0.2507	**0.0377**	0.2523
TCI - SD5	Personality trait	0.2490	**0.0391**	0.2523
Right cerebellar lobule X	Cerebellar volume	−0.2484	**0.0396**	0.2523
Right insula	Cortical volume	0.2482	**0.0398**	0.2523
Left cerebellar lobules I-II	Cortical thickness	−0.2434	**0.0439**	0.2685
Right cerebellar lobule VIIb	Cerebellar volume	−0.2430	**0.0443**	0.2685

The positive correlations are colored in red and the negative correlations in blue. Values in bold type are significant at *p*<0.05.

The Elastic Net model, trained using repeated k-fold cross-validation, showed satisfactory performance. Model quality was evaluated using the Root Mean Square Error (RMSE) and the coefficient of determination (R^2^), which were 8.129 (± 3.010) and 0.400 (± 0.233), respectively. Considering the RS-10 score range (10–70), this RMSE suggests the model can predict resilience scores with moderate accuracy.

**Figure 1:**
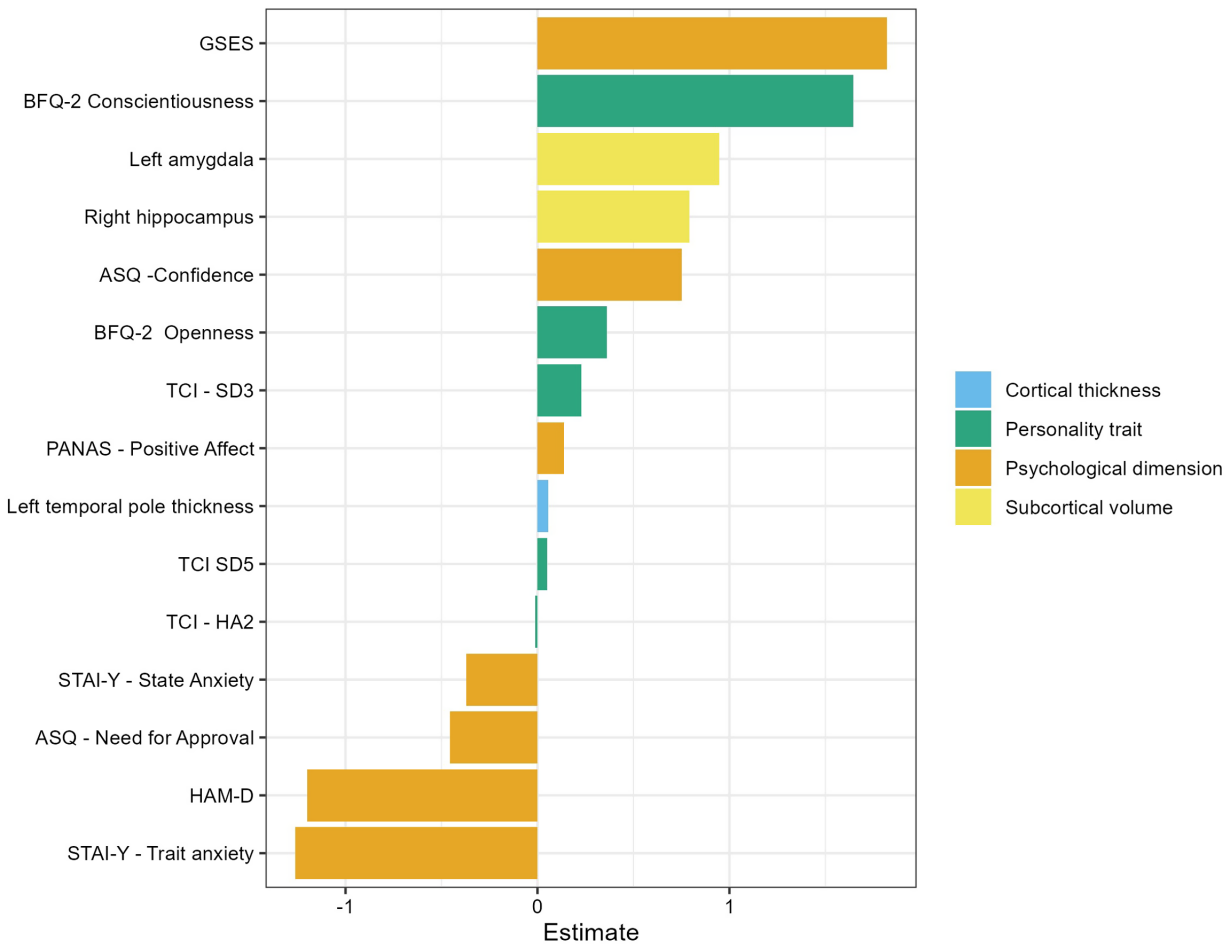
Bar plot illustrating the weight of each predictor in the Elastic Net model for resilience. Positive estimates indicate a positive association with resilience, while negative estimates indicate a negative association. Predictors are colored by type.

Fifteen variables were found to significantly predict resilience, with positive or negative estimates indicating the direction and intensity of change in the dependent variable (Figure 1). In brief, the prediction of resilience values was positively influenced by specific psychological dimensions (Self-efficacy, Confidence, Positive affect), personality traits (Conscientiousness, Openness, Resourcefulness, Enlightened second nature), and brain morphological features (amygdala and hippocampus volumes, as well as cortical thickness of the temporal pole). Conversely, the prediction of resilience values was negatively influenced by other personality traits (Fear of uncertainty) and psychological dimensions (Anxiety, Depression, Need for Approval).

Noteworthy, among the psychological factors, GSES had the greatest weight; among personality traits, the Conscientiousness factor of the BFQ-2 had the highest predictive impact; among neuro-morphological variables, the volume of the left amygdala emerged as a key predictor of resilience (Figure 1).

**Figure 2:**
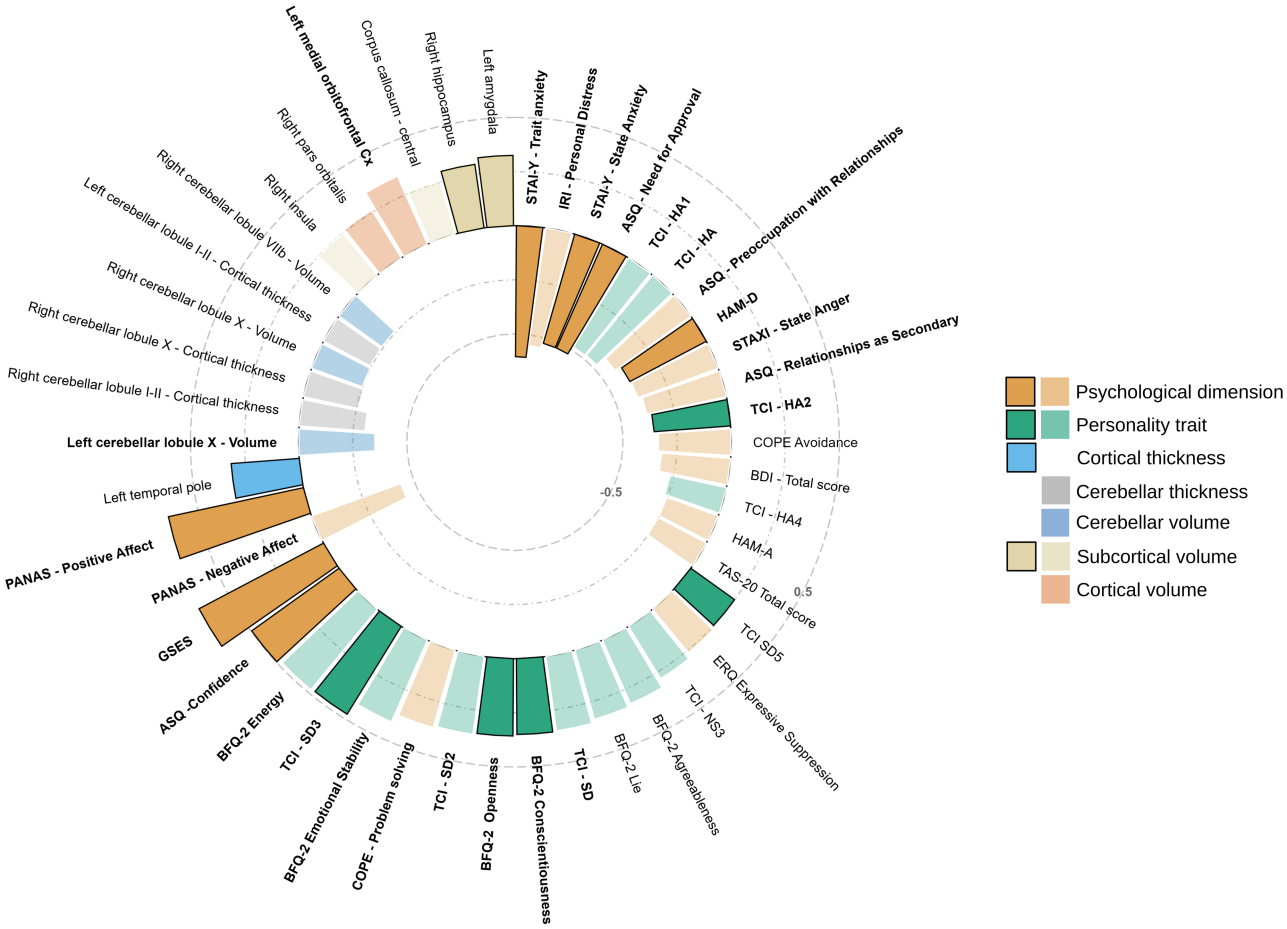
Correlations between the dependent variable RS-10 and the 46 candidate predictors of resilience scores. The figure depicts the 46 variables selected as candidate predictors for the regression. The 25 correlations that resulted significant (*p*-adjusted < 0.05) after FDR correction are written in bold, while the 15 variables that resulted as predictors are in darker colors and with an outline. The dotted concentric circles indicate the values of Spearman’s rho. TCI-HA1, TCI-Harm Avoidance Anticipatory worry; TCI-HA, TCI-Harm Avoidance; TCI-HA2, TCI-Harm Avoidance Fear of uncertainty; TCI-HA4, TCI-Harm Avoidance Fatigability; TCI-SD5, TCI-Self-directedness Enlightened second nature; TCI-NS3, TCI-Novelty Seeking Extravagance; TCI-SD, TCI-Self-directedness; TCI-SD2, TCI-Self-directedness Purposefulness; TCI-SD3, TCI-Self-directedness Resourcefulness; Cx, Cortex.

Figure 2 summarizes the ensemble of correlations between the dependent variable RS-10 and the 46 candidate predictors of resilience.

## 4 Discussion

As Feldman (2020) says, the flexible regulation and integration of multiple psychological processes and brain systems allow them not only to coexist but also to dynamically coalesce into a functionally resilient whole. In the same line but with a major emphasis on the neurobiological processes, Cathomas et al. (2019) have suggested conceptualizing resilience as a process requiring the integration of various central (hippocampal neurogenesis, dopaminergic systems, transcriptional and epigenetic pathways) and peripheral (cellular and humoral factors of the immune system, gut microbiota, blood–brain barrier) systems.

Several studies have analyzed the psycho-social factors contributing to resilience, among them emotion regulation, executive functions, dispositional optimism, coping strategies, cognitive reappraisal, and social support (Southwick and Charney, 2012). Many of these protective factors are interlinked. For example, greater emotional regulation is associated with strengthened executive functions and cognitive flexibility (Mohammed et al., 2022).

Based on this integrative approach, we provide an explorative model encompassing psychological and personality factors and cortical and subcortical morphological variables that may predict the resilient phenotype. Namely, we found that resilience was positively predicted by Self-efficacy, Confidence, Positive affect, as well as by Conscientiousness, Openness, Resourcefulness, Enlightened second nature, and by amygdala and hippocampus volumes, and temporal pole cortical thickness. Conversely, resilience was negatively influenced by Fear of uncertainty, and Anxiety, Depression, and Need for Approval.

### 4.1 Self-efficacy

The factor with the most predictive value was linked to self-efficacy by GSES, defined as individuals’ confidence in their ability to succeed in particular situations or to accomplish a task. The very definition of self-efficacy emphasizes how much it is related to resilience. By determining the beliefs people hold regarding their power to affect situations, self-efficacy strongly influences both the power to competently face challenges and the most likely made choices. People with high self-efficacy values perceive challenges as opportunities to be mastered instead of threats to be avoided (Bandura, 2006). Self-efficacy not only impacts our lives during highly stressful situations but also enhances our motivation and the capacity to pursue ambitious goals.

Although self-efficacy and resilience are distinct psychological resources, independent from each other, they are highly related since both of them share the ability to persevere in the face of difficulty and have a positive self-concept. By activating affective, motivational, and behavioral mechanisms in demanding situations, self-efficacy beliefs can promote resilience so much that sometimes self-efficacy has been conceptualized as one component of resilience (Werner, 1997). Remarkably, high self-efficacy levels have been linked to low levels of anxiety and low vulnerability to depression (Hinz et al., 2023). Note that within the predictors of resilience found in the present research, the values of anxiety and depression scales were the factors with the highest negative coefficients associated with resilience.

### 4.2 Personality

It is worth noting that within personality traits, Conscientiousness and Openness – factors of the Big Five model – and Resourcefulness and Enlightened second nature – subscales of TCI character dimension Self-directedness – predicted resilience values with positive coefficients, while Fear of uncertainty – subscale of the TCI Harm Avoidance temperamental dimension – predicted resilience values with negative coefficient. These outcomes fully agree with literature findings on healthy individuals of different ages (Graham et al., 2021; Linnemann et al., 2022; Nieto et al., 2023), although such associations have been more frequently described in patients with various pathologies. Briefly, Conscientiousness is the personality trait that implies being careful and diligent, efficient and organized. Although with less predictive impact, resilience values were also predicted by the trait Openness, which assesses how open-minded, imaginative, creative, and insightful a person is. Those who are more broadminded tend more willing to listen to multiple viewpoints or try new things (high cognitive flexibility). Not very differently, the most distinctive characteristics of self-directed individuals are that they are effective and able to adapt their behavior according to voluntary goals. Finally, we found that resilience displayed a small negative relationship with Fear of uncertainty, which is associated with behavioral inhibition, avoidance of aversive situations, poor coping, proneness to negative emotions, and the tendency to be sensitive to punishment signals. Furthermore, the TCI subscales Resourcefulness and Enlightened second nature were associated with RS-10 scores with positive coefficients. As a final note, it has to be underlined that the further subscales of TCI (Purposefulness, Extravagance, Anticipatory worry) and BFQ-2 (Energy/Extraversion, Emotional Stability) were significantly correlated to RS-10, even if not resulting as predictors in the Elastic Net model.

This ensemble of personality-related predictors of resilience reflects the benefits of having a hard-working and positive affective style as well as abilities of interpersonal closeness and social interaction. On one hand, the meticulous approach of conscientious individuals may lend itself well to effectively coping with negative life experiences, resulting in a sense of self-efficacy. On the other hand, positive emotions and close social interactions contribute to resilience because they broaden the “thought-action repertoires.”

### 4.3 Attachment

The attachment style is an acknowledged descriptor of interpersonal patterns beginning with early interactions with the primary caregiver (Bowlby, 2003). The initial experiences have a great impact over time and influence the way later relationships are processed, the ability to cope with hardships, and the overall functioning and mental health. Attachment styles have been classically categorized as secure or insecure. It has been shown that secure attachment can foster resilience by implementing effective problem-focused coping strategies, which may in turn shape resilience (Rasmussen, 2019; Villasana et al., 2016). Secure attachment and resilience are complementary concepts, which share similar developmental circumstances, stemming from a healthy childhood, and leading to the emergence of adaptive self-esteem and empathy, through positive relations with others. Actually, individuals with a secure attachment exhibit high levels of resilience, and both these variables, in turn, correlate positively with proactive coping strategies, and negatively with avoidant coping strategies (Marriner et al., 2014). Intriguingly, we found that among the predictors of resilience, there were the positively predicting ASQ dimension Confidence and the negatively predicting ASQ dimension Need for Approval. The other ASQ dimensions, Relationships as Secondary and Preoccupation with Relationships as well as the coping strategy Problem Solving assessed by COPE inventory were positively correlated with the RS-10, although not resulting as predictors in the Elastic Net model. Specifically, active coping strategies are intentional efforts aimed at minimizing the physical, psychological, or social harm of a stressor. They are associated with actual or perceived control over the stressor, leading to changes facilitating resilient responses (Wood and Bhatnagar, 2015).

### 4.4 Positive affect

Research has repeatedly demonstrated that experiencing positive emotions in the face of adversity is one of the most important processes involved in resilience (Fredrickson, 2001; Paquette et al., 2023). In accordance, we found that resilience values were associated positively with positive mood scores and negatively with negative mood scores, as assessed by PANAS. Notably, positive emotions resulted as predictors in the present Elastic Net model for resilience. This result is not surprising, given that positive emotions play the role of a buffer between the distressing situation and the emotional elicitation and appraisal of that situation (Paquette et al., 2023; Philippe et al., 2009). The role of positive emotions in resilient behaviors has been explained by the “Broaden-and-Build Theory” (Fredrickson, 2001), positing that positive emotions facilitate resilience by broadening one’s attention and effective coping strategies (Tugade et al., 2004). Repeated experiences of positive emotions would render this broadened mindset habitual and result in increased personal resources that can be drawn on in times of need and facilitate resilient behaviors and adaptive coping strategies (Tugade et al., 2004). In addition, positive emotions would have an undoing effect, given that they counteract the deleterious after-effects of negative emotions and stress (Cohn et al., 2009).

### 4.5 Anxiety and depression

Identifying the profile that characterizes resilient people and has predictive value as to whether or not anxiety and depression symptoms will be present is an important issue in the resilience literature (Wermelinger Ávila et al., 2017). In agreement with previous reports (Lyu et al., 2022; To et al., 2022), we found that state and trait anxiety, as well as depression, showed the highest negative coefficients associated with resilience. Predictably, as resilience reflects the ability to adaptively act against psychological distress, we found significant inverse relationships between resilience values and anxiety and depression scores. These negative predictors suggest that resilience resources may turn the triggers for anxiety and depression into opportunities to improve performances and overcome difficulties, accordingly with the inverse associations between resilience and psychological distress described in patients with chronic diseases (To et al., 2022), and older people (Wermelinger Ávila et al., 2017).

### 4.6 Structural brain correlates of resilience

In addition to the psychological and personality factors, we inserted several brain morphological features within the present regression model to achieve an even more multifaceted profile of the resilient phenotype.

Human cross-sectional studies have focused on neural structures and neuroendocrine markers of resilience, and the animal models provided data on the behavioral, genetic, molecular, and hormonal bases of resilience, showing that susceptible subjects exhibit specific molecular abnormalities and distinct epigenetic and cellular adaptations lacking in resilient individuals (Laricchiuta et al., 2023a,b; Nasca et al., 2019; Russo et al., 2012). In brief, the neuronal architecture of resilience largely overlaps with the neuronal structures related to cognitive and emotional regulation, as the executive control network (including prefrontal, frontal, and parietal regions) and the emotional arousal network (including cingulate cortex subregions, amygdala, hippocampus, and insula). However, up to now, these brain structures have been mainly implicated in the vulnerability, rather than in the resilience, to stress or trauma (Laricchiuta et al., 2023a,b). In fact, since the brain is continuously adapting to the perturbations in bodily homeostasis, most information regards the neurobiology of resilience as a response to disease or traumatic adversities and not to trait resilience, which we were conversely mainly interested in. However, since the maladaptive responses to stress/trauma are the flipside of resilience, it is appropriate to take into account even the literature data analyzing the brain morphological responses to stress/trauma. Interestingly, in the present model of resilience, the volumes of the amygdala and hippocampus, as well as the cortical thickness of the temporal pole, predicted the resilience values with positive coefficients.

Let us analyze the single neuronal predictors of resilience in detail.

#### 4.6.1 Amygdala

We found that the volume of the left amygdala positively predicted higher resilience scores, consistently with the larger amygdala volumes associated with increased resilience scores in healthy subjects (Gupta et al., 2017) and with the larger amygdala and OFC activation responses to stressful events, the greater the resilience (Reynaud et al., 2013). In accordance with the present findings, Morey et al. (2016) described larger left amygdala and right hippocampal volumes in resilient maltreated children.

In literature, conflicting studies reported amygdala volumes larger (Holz et al., 2017), unmodified (Woon and Hedges, 2008), or smaller (Hanson et al., 2015) in individuals who had experienced stressful social adversity. Recently, a structural connectivity study (Ohashi et al., 2019) showed that amygdala nodal efficiency was lower in resilient than in susceptible individuals to maltreatment, suggesting that the decreased efficiency of the amygdala node in propagating information throughout the network might mitigate the effects of adversities and lead to enhanced resilience. However, it has to be noted that these contradictory findings have been attributed to the amygdala vulnerability to the type, magnitude, and timing of stress. Conversely, the present research was aimed at finding the neuronal predictors of resilience in the absence of any stressful event. A finding of the present research worth emphasizing concerns the significant correlations between RS-10 scores and the amygdala, orbitofrontal cortex, temporopolar cortex, and cerebellar lobule X, all structures involved in the network engaged in the regulation of emotional states and the development of well-adapted social skills (Bachevalier and Loveland, 2006).

#### 4.6.2 Hippocampus

The second most significant positive neuronal predictor of resilience was the volume of the right hippocampus. This finding fits with the reduction of hippocampal volume repeatedly described in individuals affected by trauma-related psychopathologies or mood disorders or living in poverty (Davidson and McEwen, 2012; Kim et al., 2015; Levy-Gigi et al., 2015; MacQueen et al., 2003; Suzuki et al., 2013), given that one of the core symptoms of these conditions is the altered regulation of emotions induced by traumatic memories. Notably, animal studies report that exposure to traumatic events damages hippocampal neurons, inhibits neurogenesis, and inhibits the development of new granule neurons in the dentate gyrus (Schoenfeld and Gould, 2012). The opposite description of increased hippocampal volumes associated with increased resilience is less established (Richter et al., 2019). A study by Vermetten et al. (2003), which reports that psychopharmacological treatment of PTSD symptoms resulted in increased hippocampal volumes, suggests that larger hippocampal volumes are related to higher resilience. Similarly, pharmacological treatment with antidepressants reverses the decreased hippocampal volumes by increasing neural progenitor cells (Boldrini et al., 2012). Furthermore, the deleterious effect of poverty on hippocampal volume is alleviated in subjects with high self-esteem, suggesting that positive psychological resources may protect against hippocampal atrophy in adversity (Wang et al., 2016).

Of note, several studies found larger hippocampal volumes in resilient individuals in comparison to PTSD subjects (Kasai et al., 2008). In healthy volunteers, greater functional coupling between the hippocampus and ventromedial prefrontal cortex is suggested to be linked to greater extinction recall, a capacity thought to promote resilience (Milad et al., 2007). Subjects with high adversity levels but high resilience scores show reduced reward-related activation of the ventral striatum and increased activation of the ventral tegmental area and hippocampus (Richter et al., 2019). In conclusion, literature data support the idea that larger hippocampal volumes may be associated with resilience, as found in the present research.

#### 4.6.3 Temporal pole

A further positive predictor of resilience of the present model was the cortical thickness of the left temporal pole. Cortical thickness reflects the size, density, and arrangement of neurons, neuroglia, and nerve fibers, as well as axon and dendrite remodeling and myelination.

Because of its distributed anatomical connections with limbic structures and neocortical regions, the temporal pole has been associated with several high-level cognitive processes, such as visual processing for complex objects, face recognition, autobiographical memory, and semantic processing (Herlin et al., 2021). Moreover, it has been involved in several emotional or affective circumstances, such as recalling emotionally intense autobiographical memories or watching an emotion-inducing movie (Olson et al., 2007). Notably, based on impaired recognition of facial and musical emotions associated with atrophy of the right temporal pole (Hsieh et al., 2012), the temporal pole is argued to be a conduit for integrating visceral information, sensory representations, and memories for emotionally or socially relevant concepts (Olson et al., 2007). Such emotional processing and multimodal sensory integration may contribute to stable emotional and social behaviors essential to resilience. The temporal pole is involved in the perception and comprehension of others’ thoughts and actions (Olson et al., 2007), and it is activated during empathy and theory of mind tasks (Reniers et al., 2014). Interestingly, temporal pole volume positively correlates with scores in trait modesty (Zheng et al., 2017), associated with the motivation toward prosocial behavior, stable interpersonal relationships, and adaptive psychological functioning (Zheng et al., 2017), all components of resilience. A meta-analysis of brain volumes in subjects with PTSD reported reduced volumes of the temporal pole (Kühn and Gallinat, 2013). Consistent with previous studies in non-older groups (Gupta et al., 2017; Kahl et al., 2020), and in complete agreement with the present results, a recent study on older people reported that resilience capacities are positively related to the cortical thickness of the left temporal pole (Shikimoto et al., 2021). Thus, the morphometric features of the temporal pole may predict affective regulation and, hence, resilience, as occurs in the present Elastic Net model.

## 5 Limitations

Several limitations of the present study should be acknowledged. The cross-sectional design restricts the possibility of inferring causality or directionality in the observed associations between resilience and its psychological or neurobiological correlates. As a result, we are aware that conclusions regarding temporal dynamics or developmental pathways remain speculative and warrant investigation in longitudinal studies. Although our approach was methodologically rigorous, the modest sample size remains a notable constraint of the present research. Despite this, R^2^ and RMSE metrics indicate that the model can predict resilience scores with moderate accuracy.

As a further note, our sample consisted exclusively of healthy Italian adults, which might introduce cultural or demographic biases limiting the applicability of findings to diverse populations or clinical cohorts. Factors such as cultural norms, health status, or environmental exposures could influence resilience and its correlates, and might not be adequately captured in the present homogeneous sample.

Given these limitations, the present findings must be considered exploratory and hypothesis-generating rather than confirmatory. Future research with larger, more diverse, and longitudinal samples could validate and extend these results, incorporating replication in independent cohorts and alternative modeling strategies to enhance generalizability across populations and contexts and inferential strength.

## 6 Conclusion

The profile of resilient people proposed by the present model, on one hand encompasses what has been inventively termed the ‘ordinary magic’ (Masten, 2001) of strongly adaptive fundamental systems, such as positive personality characteristics, psychological well-being linked to high self-efficacy and conscientiousness, low anxiety and depression, secure attachment, positive emotional experience, adaptive coping, broad and affective social support. On the other hand, the resilient profile includes brain structural correlates, indicating that resilient individuals are characterized by neural substrates reflecting efficient arousal modulation and emotional/cognitive regulation in a flexible interplay with psychological and environmental factors. Interestingly, the multiple variables belonging to different domains are intertwined.

One of the main findings of this holistic model of resilience is combining multifaceted factors into a unified result so that the whole is greater than the sum of its parts.

## Data Availability

The raw data supporting the conclusions of this article will be made available by the authors, without undue reservation.

## Footnotes

^1^v5.1.3, http://surfer.nmr.mgh.harvard.edu/, accessed on 26 January 2020

^2^ https://www.volbrain.net/
