# Supplementary material for: Psychological and neuro-morphological predictors of resilience in healthy adults: the whole is more than the sum of its parts
Source: Front Neurosci. 2025 Jul 9;19:1597835. doi: 10.3389/fnins.2025.1597835 (PMC12283669; doi:10.3389/fnins.2025.1597835)
Supplement: Supplementary file 1 [file Data_Sheet_1.docx]

**LIST OF CONTENTS IN SUPPLEMENTARY MATERIALS**

- **Inclusion and exclusion criteria to MRI**
- **Psychological Instruments:**

- Toronto Alexithymia Scale (TAS-20)

- Beck’s Depression Inventory (BDI)

- Attachment Style Questionnaire (ASQ)

- Interpersonal Reactivity Index (IRI) (for Empathy)

- Emotion Regulation Questionnaire (ERQ)

- Coping Orientation to Problems and Experiences (COPE)

- State-Trait Anger Expression Inventory (STAXI)

- State-Trait Anxiety Inventory – Form Y (STAI-Y)

- Positive and Negative Affect Schedule (PANAS)

- General Self-Efficacy Scale (GSES)

- Hamilton Anxiety Rating Scale (HAM-A)

- Hamilton Depression Rating Scale (Ham-D)

- SES - Holmes and Rahe Stress Scale

- Raven's Progressive Matrices (RPM)

- **Personality Assessment Instruments**

- Big Five Questionnaire 2 (BFQ-2)

- Temperament and Character Inventory (TCI)

- **MRI Data Acquisition and Processing**
- **Table S1. Descriptive statistics for socio-demographic and psychological variables**
- **Figure S1. Bar plot illustrating the frequency distribution of RS-10 scores**
- **Table S2. List of neuromorphological variables analyzed in the correlation analyses**
- **Table S3. Correlations between the dependent variable RS-10 and the 279 variables of interest**

**______________________________________________________________________________________**

**Inclusion and exclusion criteria for MRI**

**Inclusion criteria** were age between 18 and 65 years and suitability for MRI scanning (absence of non-MRI-compatible implants or prostheses, claustrophobia, or pregnancy)

**Exclusion criteria** included (1) suspicion of cognitive impairment or dementia based on Mini Mental State Examination (MMSE)1 scores ≤ 242, and confirmed by clinical neuropsychological evaluation using the Mental Deterioration Battery (Picerni et al., 2022) and the NINCDS-ADRDA criteria for dementia (Sagone et al., 2023); (2) subjective complaint of memory difficulties or of any other cognitive deficit, regardless of interference with daily activities; (3) major medical illnesses, e.g. diabetes (not stabilized), obstructive pulmonary disease, or asthma; hematologic and oncologic disorders; pernicious anemia; clinically significant gastrointestinal, renal, hepatic, endocrine, or cardiovascular system diseases; newly treated hypothyroidism; (4) current or reported psychiatric (assessed by SCID-I and the SCID-II) (First, 1997; First et al., 1997) or neurological (assessed by clinical neurological evaluation) disorders (e.g. schizophrenia, mood disorders, anxiety disorders, stroke, Parkinson’s disease, seizure disorder, head injury with loss of consciousness, and any other significant mental or neurological disorder); (5) known or suspected history of alcoholism or drug dependence and abuse, evaluated by structured interviews (SCID I or SCID II) (First, 1997; First et al., 1997); (6) MRI evidence of focal parenchymal abnormalities or cerebro-vascular diseases: for each subject, a trained neuroradiologist and a neuropsychologist expert in neuroimaging co-inspected all the available clinical MRI sequences (i.e. T1- and T2-weighted and FLAIR images) to ensure that the subjects were free from structural brain pathologies and vascular lesions (i.e. FLAIR or T2-weighted hyper-intensities and T1-weighted hypo-intensities).

The investigation was carried out in accordance with the latest version of the Declaration of Helsinki. Both behavioral and MRI protocols were approved by the local ethics committee of the Santa Lucia Foundation IRCCS (CE/PROG.461,15/9/2014). Written informed consent of all participants was obtained before the study.

**________________________________________________________________________**

**Psychological Instruments**

In this study, participants’ psychological profile was assessed by means of the Italian versions of the following psychological questionnaires and scales:

- Attachment Style Questionnaire (ASQ);

- Beck’s Depression Inventory Scale (BDI);

- Coping Orientation to Problems and Experiences (COPE);

- Emotion Regulation Questionnaire (ERQ);

- General Self-Efficacy Scale (GSES);

- Hamilton Anxiety Rating Scale (HAM-A);

- Hamilton Depression Rating Scale (HAM-D);

- Holmes and Rahe Stressful Event Scale (HR-SS);

- Interpersonal Reactivity Index (IRI);

- Positive and Negative Affect Schedule (PANAS);

- State-Trait Anger Expression Inventory (STAXI);

- State-Trait Anxiety Inventory-Form Y (STAI-Y);

- Toronto Alexithymia Scale (TAS-20).

- Raven’s Progressive Matrices (RPM);

**Attachment Style Questionnaire (ASQ)**

The ASQ (Feeney et al., 2014; Fossati et al., 2003; Picerni et al., 2022; Sagone et al., 2023) is a self-administered questionnaire designed to measure five dimensions of adult attachment: Confidence (about themselves and others; 8 items), which is associated to secure attachment; Discomfort with Closeness (referring to the difficulty in trusting others; 10 items) and Relationships as Secondary (referring to the belief that achievement is more important than relationships with others; 7 items), which are associated to insecure/avoidant attachment; Need for Approval (referring to the focus on validation from others and fear of rejection and avoiding doing things that other people will not like; 7 items) and Preoccupation with Relationships (referring to the worry of being abandoned and not making it on your own; 8 items), which are associated to insecure/anxious attachment (Bartholomew & Horowitz, 1991; Hazan & Shaver, 1987). Each item is rated on a 6-point Likert-type scale from 1= totally disagree to 6= totally agree, in which the participants indicate the degree to which the propositions describe their feelings.

**Beck’s Depression Inventory (BDI)**

The BDI is a 21-item multiple-choice self-report inventory rated on a 4-point scale ranging from 0 to 3 that assesses the presence of depressive symptoms (Beck et al., 1988; Laricchiuta et al., 2015). Scores can range from 0 to 63, with higher scores indicating more severe depression. The total score is defined as the sum of the individual item scores. The questionnaire is composed of items relating to symptoms of depression, such as hopelessness and irritability, beliefs, such as guilt or feelings of being punished, as well as physical symptoms such as fatigue, weight loss, and lack of interest in sex.

**Coping Orientation to Problems and Experiences (COPE)**

The COPE is a 60-item self-report multidimensional questionnaire designed to measure different strategies generally used to cope with stressful life events (Carver et al., 1989; Laricchiuta et al., 2022; Peveri, 2010; Sica et al., 1997, 2008). “Coping” is defined broadly as an effort used to minimize distress associated with negative life experiences. Items are rated on a 4-point Likert scale ranging from 1 = *usually don’t do this at all* to 4 = *usually do this a lot*. The test consists of five subscales: Social support - referring to the search for sharing opportunities, information, and emotional outbursts; Avoidance - referring to denial, behavioral and mental detachment, and substance abuse; Positive attitude - referring to the attitude of acceptance, containment and positive reinterpretation of events; Problem solving - referring to the use of active strategies and planning; Turning to religion - referring to religion and the absence of humor. These subscales measure substantially independent dimensions, and proved to be reliable and fairly stable in time (Sica et al., 2008).

**Emotion Regulation Questionnaire (ERQ)**

The ERQ (Balzarotti et al., 2010; Gross & John, 2003; Laricchiuta et al., 2022) is a self-report measure made of 10 items, scored on a 7-point Likert scale ranging from 1 = *strongly disagree* to 7 = *strongly agree.* It is composed of two subscales assessing individual differences in the habitual use of two different emotion regulation strategies: Cognitive Reappraisal (6 items) and Expressive Suppression (4 items). Namely, Cognitive Reappraisal is an antecedent-focused strategy, affecting the early cognitive stages of emotional activity, in which individuals attempt to modulate the emotional salience of a stimulus by changing its meaning (Gross, 1998). It is considered effective and adaptive (especially when individuals face uncontrollable stress) since it is often correlated positively with better mental and physical health, positive affect, and extraversion, and negatively with negative affect, neuroticism, and depressive symptoms (Aldao et al., 2015; McRae & Gross, 2020). By contrast, Expressive Suppression is a response-focused strategy implemented after the emotion has been generated and it is conceptualized as preventing outward expression of internal emotional state, without modifying emotional experience and arousal (Gross, 1998). Although suppression may have short-term benefits (Goldin et al., 2008), it can be considered maladaptive since it is often correlated negatively with physical and psychological health positive affect, extraversion, and relationship satisfaction, and positively with psychopathology (McRae & Gross, 2020). Higher scores on a given subscale indicate a greater tendency to use that emotional regulation strategy.

**General self-efficacy scale (GSES)**

The GSES is a 10-item self-report measuring the general self-efficacy which refers to the belief in one’s competence to cope with unexpected new experiences or life’s adversities (Luszczynska et al., 2005; Peveri, 2010; Schwarzer et al., 1995). Items are rated on a 4-point Likert scale ranging from 1 = *strongly disagree* to 4 = *strongly agree*. The total score, calculated by finding the sum of all items, ranges between 10 and 40, with a higher score indicating more self-efficacy.

**Hamilton Anxiety Rating Scale (HAM-A)**

The presence and severity of anxiety symptoms were evaluated by using HAM-A (Hamilton, 1959; Picerni et al., 2021), which consists of 14 items, each defined by a series of symptoms, and measures both psychic anxiety (mental agitation and psychological distress) and somatic anxiety (physical complaints related to anxiety). Each item is scored on a scale of 0 = *not present* to 4 = *severe*, with total scores ranging from 0 to 56. Scores < 5 indicate no anxiety, scores between 6 and 13 indicate mild anxiety, and scores > 14 indicate moderate to severe anxiety.

**Hamilton Depression Rating Scale (Ham-D)**

The presence and severity of depressive symptoms were evaluated by using HAM-D (Hamilton, 1960; Picerni et al., 2021), which contains 17 items. It is used to rate depression severity by probing [mood](https://en.wikipedia.org/wiki/Mood_(psychology)), feelings of guilt, suicide ideation, [insomnia](https://en.wikipedia.org/wiki/Insomnia), [agitation](https://en.wikipedia.org/wiki/Psychomotor_agitation), retardation, [anxiety](https://en.wikipedia.org/wiki/Anxiety), [weight loss](https://en.wikipedia.org/wiki/Weight_loss), and somatic symptoms. Each item is scored on a 3- or 5-point scale, depending on the item. The maximum score is 52. Total scores < 7 indicate no depression, scores from 8 to 17 correspond to mild depression, scores from 18 to 24 correspond to moderate depression, and scores > 25 severe depression.

**Holmes and Rahe Stress Scale (HR-SS)**

The HR-SS (Holmes & Rahe, 1967) provides a list of 43 stressful life events that have dramatically changed a person’s life. We asked participants to refer to the events that occurred in the last 3 months. Higher scores reflect the respondents’ higher levels of stress.

**Interpersonal Reactivity Index (IRI)**

The IRI is a self-report multidimensional measure of trait empathy (Albiero et al., 2006; Chrysikou & Thompson, 2016; Davis, 1980; Davis, 1983; Picerni et al., 2021). The questionnaire is based on a self-report comprising 28 items answered on a 5-point Likert scale ranging from 1 = *never true for me* to 5 = *always true for me*. The measure has 4 subscales, each made up of 7 different items, and for each subscale, scores can range from 7 to 35. These subscales are: Perspective Taking, which indicates the tendency to spontaneously adopt the psychological point of view of others; Fantasy, which taps respondents' tendencies to transpose themselves imaginatively into the feelings and actions of fictitious characters in books, movies, and plays; Empathic Concern, which assesses "other-oriented" feelings of sympathy and concern for unfortunate others; Personal Distress, which measures "self-oriented" feelings of personal anxiety and unease in tense interpersonal settings.

**Positive and Negative Affect Schedule (PANAS)**

The PANAS (Peveri, 2010; Terracciano et al., 2003; Watson et al., 1988) measures someone’s positive and negative affect. Participants are required to indicate on a 5-point Likert scale to what extent (1 = *very slightly*, 5 = *extremely*) they generally experienced 20 different affective states described by adjectives (10 for positive affect – such as “Interested”, “Proud”, etc. - and 10 for negative affect - “Hostile”, Upset”, etc.). Scores can range from 10 to 50 for both Positive and Negative Affect, with the lower/higher scores representing lower/higher levels of Positive/Negative Affect.

**State-Trait Anger Expression Inventory (STAXI)**

The STAXI (Spielberger, 2021) measures the experience of anger conceptualized as having two major components: state and trait anger. State anger is defined as a psychobiological emotional state or condition marked by subjective feelings varying in intensity from mild irritation or annoyance to intense fury and rage, and generally accompanied by muscular tension and by arousal of the neuroendocrine and autonomic nervous systems. Trait anger is defined in terms of individual differences in the disposition to perceive a wide range of situations as annoying or frustrating and by the tendency to respond to such situations with elevations in state anger. Individuals with high trait anger experience state anger more often and with greater intensity than individuals who are low in trait anger. The STAXI questionnaire consists of 44 items, grouped on two main scales, State anger and Trait anger, as well as an Anger Expression Index, which tends to be most closely associated with hypertension.

**State-Trait Anxiety Inventory – Form Y (STAI-Y)**

The STAI-Y is a self-report inventory that measures state and trait anxiety (Barcaccia et al., 2018; Laricchiuta et al., 2022; Pedrabissi & Santinello, 1989; Spielberger et al., 1983). Form Y contains 20 items that assess state anxiety (STAI-Y1) and 20 items for trait anxiety (STAI-Y2). For state anxiety items, participants are asked to indicate whether the statement describes their current mood state. Responses are based on a 4-point scale ranging from 1 = *not at all* to 4 = *extremely*. For trait anxiety items, participants indicate whether the statement describes how they feel most of the time. Responses are based on a 4-point scale ranging from 1= *almost never* to 4 = *almost always*. Scores can range from 20 to 80 for each subscale.

**Toronto Alexithymia Scale (TAS-20)**

The TAS-20 is a 20-item self-report scale that measures alexithymia, that is, the difficulty in identifying and describing the experienced [emotions](https://en.wikipedia.org/wiki/Emotion) by oneself or others (Bagby et al., 1994; Laricchiuta et al., 2015). The scale has 3 subscales: Difficulty in Describing Feelings (F1; 5 items), Difficulty in Identifying (F2; 7 items), Externally Oriented Thinking (F3; 8 items). Items are scored using a 5-point Likert scale whereby 1 = *strongly disagree* and 5 = *strongly agree*. While the score for each subscale is the sum of the responses to that subscale, the total score is the sum of responses to all 20 items. TAS-20 total score can range from 20 to 100. The TAS-20 uses cutoff scoring: equal to or less than 50 = non-alexithymia; from 51 to 60 = possible alexithymia; equal to or greater than 61 = alexithymia.

**Raven’s progressive matrices (RPM)**

Raven's 47 progressive matrices is a non-verbal test typically used to measure general human intelligence and abstract reasoning ability and is regarded as a non-verbal estimate of fluid intelligence (Raven, 1936). It is included in Mental Deterioration Battery (Carlesimo et al., 1996) and comprises 60 multiple-choice questions, listed in order of increasing difficulty. In each test item, the subject is asked to identify the missing element that completes a pattern. RPM 47 provides two scores, a raw score and a converted age-based corrected score.

**________________________________________________________________________**

**Personality Assessment Instruments**

Participants’ **personality traits** were assessed by means of the Italian versions of the following tests:

- Big Five Questionnaire-2 (BFQ-2);

- Temperament and Character Inventory (TCI).

**Big Five Questionnaire-2 (BFQ-2)**

The BFQ-2 (Caprara et al., 2008) is a 134-item self-report instrument, with items rated on a 5-point Likert scale ranging from complete disagreement (1 = *absolutely false for me*) to complete agreement (5 = *absolutely true for me*). The BFQ-2 was developed to operationalize the personality dimensions of the Big Five model (Costa & McCrae, 1990), namely Energy/Extraversion (*outgoing/energetic vs. solitary/reserved*), Agreeableness (*friendly/compassionate vs. analytical/detached*), Conscientiousness (*efficient/organized vs. easy‑going/careless*), Emotional Stability (*sensitive/nervous vs. secure/confident*), and Openness (*inventive/curious vs. consistent/cautious*). Each personality dimension is divided into two sub‑dimensions (Energy/Extraversion: Dynamism and Dominance; Agreeableness: Cooperativeness and Politeness; Conscientiousness: Scrupulousness and Perseverance; Emotional Stability: Emotion control and Impulse control; Openness: Openness to culture and Openness to experience) and assessed through 24 items (12 items for each sub-dimension). The BFQ-2 also provides a score to a Lie scale aimed at assessing socially desirable responses. It consists of two sub-dimensions (Lie egoistic and Lie moralistic) assessed through 14 items (7 items for each sub-dimension). The present study considers the five Big Five dimensions and the Lie scale.

**Temperament and Character Inventory (TCI)**

TCI by Cloninger (Cloninger et al., 1993; Laricchiuta et al., 2014; Laricchiuta et al., 2014) is a 240-item self-administered dimensional questionnaire designed to evaluate the 7 (4 temperament + 3 character dimensions measured by subscales) basic dimensions.

The 4 temperament dimensions are: *i)* Novelty seeking (NS; *subscales:* Exploratory excitability (NS1), Impulsiveness (NS2), Extravagance (NS3), Disorderliness (NS4)) which refers to a tendency to exploratory activity in response to novelty, impulsive decision making, extravagant approach to cues of reward, and quick loss of temper; *ii)* Harm avoidance (HA; *subscales:* Anticipatory worry (HA1), Fear of uncertainty (HA2), Shyness with strangers (HA3), Fatigability (HA4)) refers to a tendency to inhibit behaviors and act with caution, apprehensiveness, and pessimism, to respond intensively to aversive stimuli; *iii)* Reward dependence (RD; *subscales:* Sentimentality (RD1), Attachment (RD3), Dependence (RD4)) which refers to a tendency to maintain ongoing behaviors previously associated with reinforcement and to express social attachment and dependence on the others’ approval; *iv)* Persistence (P) which refers to the ability to maintain arousal and motivation internally in the absence of immediate external reward.

The character dimensions are: *i)* Self-directedness (SD; *subscales:* Responsibility (SD1), Purposefulness (SD2), Resourcefulness (SD3), Self-acceptance (SD4), Enlightened second nature (SD5)) which refers to the executive ability of an individual to control, regulate, and adapt behavior to fit the situation in accordance to personal goals; *ii)* Cooperativeness (C; *subscales:* Social acceptance (C1), Empathy (C2), Helpfulness (C3), Compassion (C4), Pure-hearted conscience (C5)) which accounts for individual differences in the acceptance of other people; *iii)* Self-transcendence (ST; *subscales:* Self-forgetful (ST1), Transpersonal identification (ST2), Spiritual acceptance (ST3)) which is viewed as the identification with everything conceived as essential and consequential parts of a unified whole (Cloninger et al., 1993, 1994).

**________________________________________________________________________**

**MRI Data Acquisition and Processing**

Participants underwent a neuroimaging protocol including standard clinical sequences (FLAIR, DP-T2-weighted) and a volumetric whole-brain 3D high-resolution T1-weighted sequence, performed with a 3 T Allegra MR imager (Siemens, Erlangen, Germany), with a standard quadrature head coil. Volumetric whole-brain T1-weighted images were obtained in the sagittal plane using a Modified Driven Equilibrium Fourier Transform (MDEFT) sequence (Echo Time/Repetition Time-TE/TR- = 2.4/7.92 ms, flip angle 15, voxel size 1 x 1 x 1 mm^3^). All planar sequence acquisitions were obtained in the plane of the anterior-posterior commissure line.

MRI-based quantification of brain regions was performed using Freesurfer (v5.1.3, http://surfer.nmr.mgh.harvard.edu/, accessed on 26 January 2020) software package (http://surfer.nmr.mgh.harvard.edu). The stream consists of five different stages, fully described elsewhere (Dale et al., 1999; Fischl & Dale, 2000; Laricchiuta et al., 2022; Laricchiuta et al., 2014). Initially, the MRI volumes were registered to the Talairach space, and the output images were intensity normalized. At the next stage, the skull was automatically stripped off the 3D anatomical data set by using a hybrid method that uses both watershed algorithms and deformable surface models. At this stage, manual intervention is needed to visualize and edit areas of the skull and the areas of the cortex or cerebellum that should be corrected. After skull stripping, the output brain mask was labeled using a probabilistic atlas (Destrieux et al., 2010) where each voxel in the normalized brain mask volume was assigned one of the following labels: cerebral WM, cerebral cortex, lateral ventricle, inferior lateral ventricle, cerebellar WM, cerebellar Cx, thalamus, caudate, putamen, pallidum, hippocampus, amygdala, accumbens area, third ventricle, fourth ventricle, brainstem, and cerebrospinal fluid. Volumetric data of definite structures were then extracted (in mm^3^) using specific algorithms, which count the number of voxels inside the structure and multiple it by the resolution of the MRI image. FreeSurfer measures were corrected for intracranial volume (ICV).

The cortical surface of each hemisphere was inflated to an average spherical surface to locate both the pial surface and the WM/GM boundary. Preprocessed images were visually inspected before including into subsequent statistical analyses. Any topological defects were excluded from the subsequent analyses.

Cortical thickness was measured based on the shortest distance between the pial surface and the GM/WM boundary at each point across the cortical mantle. The regional thickness value at each vertex for each participant was mapped to the surface of an average spherical surface using automated parcellation in FreeSurfer (Fischl et al., 2004). Segmentations of 68 (34 left and 34 right) cortical gray matter regions based on the Desikan–Killiany atlas (Desikan et al., 2006) and two whole-hemisphere measures were visually inspected and statistically evaluated for outliers.

Cerebellum parcellation was performed through a freely available patch-based multi-atlas segmentation tool called CERES (CEREbellum Segmentation) able to automatically parcellate the cerebellum lobules. CERES (Romero et al., 2017) is part of broader software pipeline for volumetric brain analysis, namely volBrain ([https://www.volbrain.net/](https://www.volbrain.net/)). The segmentation pipeline consists of a preprocessing step (denoising, linear registration to the MNI space, inhomogeneity correction, cropping, non-linear registration and local intensity normalization) and a labeling step which results in 24 volumetric and cortical thickness GM measurements, reflecting the twelve bilateral lobule values for I–II, III, IV, V, VI, crus I, crus II, VIIB, VIIIA, VIIIB, IX, and X. VolBrain data were corrected for ICV.

**REFERENCES**

Albiero, P., Ingoglia, S., & Lo Coco, A. (2006). Contributo all’adattamento italiano dell’Interpersonal Reactivity Index. *Testing Psicometria Metodologia*, *13*(2), 107–125.

Aldao, A., Sheppes, G., & Gross, J. J. (2015). Emotion regulation flexibility. *Cognitive Therapy and Research*, *39*(3), 263–278. https://doi.org/10.1007/s10608-014-9662-4

Bagby, R. M., Parker, J. D., & Taylor, G. J. (1994). The twenty-item Toronto Alexithymia Scale—I. Item selection and cross-validation of the factor structure. *Journal of Psychosomatic Research*, *38*(1), 23–32. https://doi.org/10.1016/0022-3999(94)90005-1

Balzarotti, S., John, O., & Gross, J. (2010). An Italian Adaptation of the Emotion Regulation Questionnaire. *European Journal of Psychological Assessment*, *26*. https://doi.org/10.1027/1015-5759/a000009

Barcaccia, B., Balestrini, V., Saliani, A. M., Baiocco, R., Mancini, F., & Schneider, B. H. (2018). Dysfunctional eating behaviors, anxiety, and depression in Italian boys and girls: The role of mass media. *Revista Brasileira De Psiquiatria (Sao Paulo, Brazil: 1999)*, *40*(1), 72–77. https://doi.org/10.1590/1516-4446-2016-2200

Bartholomew, K., & Horowitz, L. M. (1991). Attachment styles among young adults: A test of a four-category model. *Journal of Personality and Social Psychology*, *61*(2), 226–244. https://doi.org/10.1037/0022-3514.61.2.226

Beck, A. T., Steer, R. A., & Carbin, M. G. (1988). Psychometric properties of the Beck Depression Inventory: Twenty-five years of evaluation. *Clinical Psychology Review*, *8*(1), 77–100. https://doi.org/10.1016/0272-7358(88)90050-5

Caprara, G. V., Barbaranelli, C., Borgogni, L., & Vecchione, M. (2008). BFQ-2. *Big Five Questionnaire*, *2*. https://galileo-prod-static.s3.eu-west-1.amazonaws.com/descargas/chile/BFQ-2_CL.pdf

Carlesimo, G. A., Caltagirone, C., & Gainotti, G. (1996). The Mental Deterioration Battery: Normative data, diagnostic reliability and qualitative analyses of cognitive impairment. The Group for the Standardization of the Mental Deterioration Battery. *European Neurology*, *36*(6), 378–384. https://doi.org/10.1159/000117297

Carver, C. S., Scheier, M. F., & Weintraub, J. K. (1989). Assessing coping strategies: A theoretically based approach. *Journal of Personality and Social Psychology*, *56*(2), 267–283. https://doi.org/10.1037//0022-3514.56.2.267

Chrysikou, E. G., & Thompson, W. J. (2016). Assessing Cognitive and Affective Empathy Through the Interpersonal Reactivity Index: An Argument Against a Two-Factor Model. *Assessment*, *23*(6), 769–777. https://doi.org/10.1177/1073191115599055

Cloninger, C. R., Przybeck, T. R., Svrakic, D. M., & Wetzel, R. D. (1994). *The Temperament and Character Inventory (TCI): A guide to its development and use*. https://www.researchgate.net/profile/Robert-Cloninger/publication/264329741_TCI-Guide_to_Its_Development_and_Use/links/53d8ec870cf2e38c6331c2ee/TCI-Guide-to-Its-Development-and-Use.pdf

Cloninger, C. R., Svrakic, D. M., & Przybeck, T. R. (1993). A psychobiological model of temperament and character. *Archives of General Psychiatry*, *50*(12), 975–990. https://doi.org/10.1001/archpsyc.1993.01820240059008

Costa, P., & McCrae, R. (1990). Personality Disorders and The Five-Factor Model of Personality. *Journal of Personality Disorders*, *4*, 362–371. https://doi.org/10.1521/pedi.1990.4.4.362

Dale, A. M., Fischl, B., & Sereno, M. I. (1999). Cortical Surface-Based Analysis. *NeuroImage*, *9*(2), Article 2. https://doi.org/10.1006/nimg.1998.0395

Davis, M. (1980). A Multidimensional Approach to Individual Differences in Empathy. *JSAS Catalog Sel. Doc. Psychol.*, *10*.

Davis, M. H. (1983). Measuring individual differences in empathy: Evidence for a multidimensional approach. *Journal of Personality and Social Psychology*, *44*(1), 113–126. https://doi.org/10.1037/0022-3514.44.1.113

Desikan, R. S., Ségonne, F., Fischl, B., Quinn, B. T., Dickerson, B. C., Blacker, D., Buckner, R. L., Dale, A. M., Maguire, R. P., Hyman, B. T., Albert, M. S., & Killiany, R. J. (2006). An automated labeling system for subdividing the human cerebral cortex on MRI scans into gyral based regions of interest. *NeuroImage*, *31*(3), 968–980. https://doi.org/10.1016/j.neuroimage.2006.01.021

Destrieux, C., Fischl, B., Dale, A., & Halgren, E. (2010). Automatic parcellation of human cortical gyri and sulci using standard anatomical nomenclature. *NeuroImage*, *53*(1), 1–15. https://doi.org/10.1016/j.neuroimage.2010.06.010

Feeney, J. A., Noller, P., & Hanrahan, M. (2014). *Attachment Style Questionnaire* [Dataset]. https://doi.org/10.1037/t29439-000

First, M. B. (1997). Structured Clinical Interview for DSM-IV Axis I Disorders (SCID-I), Clinician Version (Administration Booklet). American Psychiatric Publishing, Inc. *(No Title)*. https://cir.nii.ac.jp/crid/1370846644342763406

First, M. B., Gibbon, M., Spitzer, R. L., Williams, J. B. W., & Benjamin, L. S. (1997). Structured clinical interview for DSM-IV Axis II personality disorders (SCID-II) American Psychiatric Press. *Washington, DC*, *1997*.

Fischl, B., & Dale, A. M. (2000). Measuring the thickness of the human cerebral cortex from magnetic resonance images. *Proceedings of the National Academy of Sciences*, *97*(20), Article 20. https://doi.org/10.1073/pnas.200033797

Fischl, B., van der Kouwe, A., Destrieux, C., Halgren, E., Ségonne, F., Salat, D. H., Busa, E., Seidman, L. J., Goldstein, J., Kennedy, D., Caviness, V., Makris, N., Rosen, B., & Dale, A. M. (2004). Automatically parcellating the human cerebral cortex. *Cerebral Cortex (New York, N.Y.: 1991)*, *14*(1), 11–22. https://doi.org/10.1093/cercor/bhg087

Fossati, A., Feeney, J. A., Donati, D., Donini, M., Novella, L., Bagnato, M., Acquarini, E., & Maffei, C. (2003). On the Dimensionality of the Attachment Style Questionnaire in Italian Clinical and Nonclinical Participants. *Journal of Social and Personal Relationships*, *20*(1), 55–79. https://doi.org/10.1177/02654075030201003

Goldin, P. R., McRae, K., Ramel, W., & Gross, J. J. (2008). The Neural Bases of Emotion Regulation: Reappraisal and Suppression of Negative Emotion. *Biological Psychiatry*, *63*(6), 577–586. https://doi.org/10.1016/j.biopsych.2007.05.031

Gross, J. J. (1998). Antecedent- and response-focused emotion regulation: Divergent consequences for experience, expression, and physiology. *Journal of Personality and Social Psychology*, *74*(1), 224–237. https://doi.org/10.1037//0022-3514.74.1.224

Gross, J. J., & John, O. P. (2003). Individual differences in two emotion regulation processes: Implications for affect, relationships, and well-being. *Journal of Personality and Social Psychology*, *85*(2), 348–362. https://doi.org/10.1037/0022-3514.85.2.348

Hamilton, M. (1959). The assessment of anxiety states by rating. *The British Journal of Medical Psychology*, *32*(1), 50–55. https://doi.org/10.1111/j.2044-8341.1959.tb00467.x

Hamilton, M. (1960). A rating scale for depression. *Journal of Neurology, Neurosurgery, and Psychiatry*, *23*(1), 56–62. https://doi.org/10.1136/jnnp.23.1.56

Hazan, C., & Shaver, P. (1987). Romantic love conceptualized as an attachment process. *Journal of Personality and Social Psychology*, *52*(3), 511–524. https://doi.org/10.1037/0022-3514.52.3.511

Holmes, T. H., & Rahe, R. H. (1967). The social readjustment rating scale. *Journal of Psychosomatic Research*, *11*(2), 213–218. https://doi.org/10.1016/0022-3999(67)90010-4

Laricchiuta, D., Petrosini, L., Picerni, E., Cutuli, D., Iorio, M., Chiapponi, C., Caltagirone, C., Piras, F., & Spalletta, G. (2015). The embodied emotion in cerebellum: A neuroimaging study of alexithymia. *Brain Structure & Function*, *220*(4), 2275–2287. https://doi.org/10.1007/s00429-014-0790-0

Laricchiuta, D., Petrosini, L., Piras, F., Cutuli, D., Macci, E., Picerni, E., Chiapponi, C., Caltagirone, C., & Spalletta, G. (2014). Linking novelty seeking and harm avoidance personality traits to basal ganglia: Volumetry and mean diffusivity. *Brain Structure and Function*, *219*(3), Article 3. https://doi.org/10.1007/s00429-013-0535-5

Laricchiuta, D., Petrosini, L., Piras, F., Macci, E., Cutuli, D., Chiapponi, C., Cerasa, A., Picerni, E., Caltagirone, C., Girardi, P., Tamorri, S. M., & Spalletta, G. (2014). Linking novelty seeking and harm avoidance personality traits to cerebellar volumes: Personality Traits and Cerebellar Volumes. *Human Brain Mapping*, *35*(1), Article 1. https://doi.org/10.1002/hbm.22174

Laricchiuta, D., Termine, A., Fabrizio, C., Passarello, N., Greco, F., Piras, F., Picerni, E., Cutuli, D., Marini, A., Mandolesi, L., Spalletta, G., & Petrosini, L. (2022). Only Words Count; the Rest Is Mere Chattering: A Cross-Disciplinary Approach to the Verbal Expression of Emotional Experience. *Behavioral Sciences*, *12*(8), Article 8. https://doi.org/10.3390/bs12080292

Luszczynska, A., Scholz, U., & Schwarzer, R. (2005). The general self-efficacy scale: Multicultural validation studies. *The Journal of Psychology*, *139*(5), 439–457. https://doi.org/10.3200/JRLP.139.5.439-457

McRae, K., & Gross, J. J. (2020). Emotion regulation. *Emotion (Washington, D.C.)*, *20*(1), 1–9. https://doi.org/10.1037/emo0000703

Pedrabissi, L., & Santinello, M. (1989). Verifica della validità dello STAI forma Y di Spielberger. *Giunti Organizzazioni Speciali*. https://psycnet.apa.org/record/1991-73411-001

Peveri, L. (2010). *Resilienza e regolazione delle emozioni. Un approccio multimodale*. https://boa.unimib.it/handle/10281/7893

Picerni, E., Laricchiuta, D., Piras, F., Petrosini, L., Spalletta, G., & Cutuli, D. (2022). Cerebellar engagement in the attachment behavioral system. *Scientific Reports*, *12*(1), Article 1. https://doi.org/10.1038/s41598-022-17722-x

Picerni, E., Laricchiuta, D., Piras, F., Vecchio, D., Petrosini, L., Cutuli, D., & Spalletta, G. (2021). Macro- and micro-structural cerebellar and cortical characteristics of cognitive empathy towards fictional characters in healthy individuals. *Scientific Reports*, *11*(1), Article 1. https://doi.org/10.1038/s41598-021-87861-0

Raven, J. C. (1936). *The Performances of Related Individuals in Tests Mainly Educative and Mainly Reproductive Mental Tests Used in Genetic Studies*. University of London (King’s College).

Romero, J. E., Coupé, P., Giraud, R., Ta, V.-T., Fonov, V., Park, M. T. M., Chakravarty, M. M., Voineskos, A. N., & Manjón, J. V. (2017). CERES: A new cerebellum lobule segmentation method. *NeuroImage*, *147*, 916–924. https://doi.org/10.1016/j.neuroimage.2016.11.003

Sagone, E., Commodari, E., Indiana, M. L., & La Rosa, V. L. (2023). Exploring the Association between Attachment Style, Psychological Well-Being, and Relationship Status in Young Adults and Adults-A Cross-Sectional Study. *European Journal of Investigation in Health, Psychology and Education*, *13*(3), 525–539. https://doi.org/10.3390/ejihpe13030040

Schwarzer, R., Jerusalem, M., Weinman, J., Wright, S., & Johnston, M. (1995). Generalized Self-Efficacy Scale. *Measures in Health Psychology: A User’s Portfolio. Causal and Control Beliefs Windsor*.

Sica, C., Magni, C., Ghisi, M., Altoè, G., Sighinolfi, C., Chiri, L., & Franceschini, S. (2008). Coping Orientation to the Problems Experiences-new Italian version (COPE-NVI). *Psicoterapia Cognitiva e Comportamentale*, *14*, 27–53.

Sica, C., Novara, C., Dorz, S., & Sanavio, E. (1997). Coping Orientation to Problems Experienced (COPE): Traduzione e adattamento italiano. *Giunti Organizzazioni Speciali*. https://psycnet.apa.org/record/1998-01058-002

Spielberger, C. (2021). *State-Trait Anger Expression Inventory* [Dataset]. https://doi.org/10.1037/t29496-000

Spielberger, C., Gorsuch, R., Lushene, R., Vagg, P., & Jacobs, G. (1983). Manual for the State-Trait Anxiety Inventory (Form Y1 – Y2). In *Palo Alto, CA: Consulting Psychologists Press; Vol. IV*.

Terracciano, A., McCrae, R. R., & Costa, P. T. (2003). Factorial and construct validity of the Italian Positive and Negative Affect Schedule (PANAS). *European Journal of Psychological Assessment: Official Organ of the European Association of Psychological Assessment*, *19*(2), 131–141. https://doi.org/10.1027//1015-5759.19.2.131

Watson, D., Clark, L. A., & Tellegen, A. (1988). Development and validation of brief measures of positive and negative affect: The PANAS scales. *Journal of Personality and Social Psychology*, *54*(6), 1063–1070. https://doi.org/10.1037//0022-3514.54.6.1063

**______________________________________________________________________________**

**Table S1. Descriptive statistics for socio-demographic and psychological variables.**

| **Socio-demographic variables** | | **Females** | | **Males** | |
| --- | --- | --- | --- | --- | --- |
|  |  |  | |  | |
| **Sex** |  | 41 | | 28 | |
|  | | **mean** | **SD** | **mean** | **SD** |
| **Age** |  | 41.88 | 12.69 | 38.21 | 11.87 |
| **Educational Level** |  | 15.68 | 2.73 | 15.86 | 3.37 |
|  | | | | | |
| **Psychological tests** | **Subscales** | **mean** | **SD** | **mean** | **SD** |
| **RS-10 (Resilience)** |  | 55.22 | 11.36 | 58.79 | 7.49 |
| **ASQ (Attachment)** | Confidence | 34.51 | 5.07 | 33.86 | 4.27 |
|  | Discomfort with Closeness | 34.85 | 7.15 | 33.71 | 6.59 |
|  | Relationships as Secondary | 14.17 | 5.27 | 15.75 | 4.27 |
|  | Need for Approval | 19.37 | 5.76 | 18.25 | 5.97 |
|  | Preoccupation with Relationships | 24.98 | 5.66 | 25.50 | 7.38 |
| **BDI (Depression)** | Total Score | 4.71 | 4.03 | 4.04 | 4.18 |
| **COPE (Coping Strategies)** | Social Support | 34.20 | 6.13 | 31.36 | 7.74 |
|  | Avoidance | 26.63 | 5.45 | 25.07 | 4.59 |
|  | Positive Attitude | 34.54 | 4.14 | 32.57 | 4.20 |
|  | Problem Solving | 34.78 | 4.51 | 35.50 | 4.26 |
|  | Turning to Religion | 21.39 | 4.77 | 18.11 | 5.06 |
| **ERQ (Emotional regulation strategies)** | Cognitive Reappraisal | 5.35 | 0.93 | 4.88 | 0.95 |
|  | Expressive Suppression | 3.16 | 1.26 | 3.86 | 0.94 |
| **GSES (Self-efficacy)** |  | 29.17 | 4.89 | 30.36 | 4.10 |
| **HAM-A (Anxiety)** |  | 5.54 | 3.82 | 4.07 | 3.91 |
| **HAM-D (Depression)** |  | 3.37 | 2.75 | 2.36 | 2.93 |
| **HR-SS (Stressful events)** |  | 99.76 | 89.90 | 100.43 | 100.32 |
| **IRI (Empathy)** | Perspective Taking | 25.85 | 3.29 | 24.64 | 3.70 |
|  | Fantasy | 22.80 | 4.41 | 20.04 | 5.43 |
|  | Empathic Concern | 27.83 | 3.73 | 25.07 | 3.27 |
|  | Personal Distress | 17.88 | 4.73 | 15.14 | 3.74 |
| **PANAS (Positive and negative affect)** | Positive Affect | 36.63 | 5.97 | 37.25 | 4.36 |
|  | Negative Affect | 21.63 | 6.28 | 21.75 | 7.19 |
| **STAXI (Anger)** | State Anger | 10.49 | 2.64 | 10.14 | 0.45 |
|  | Trait Anger | 16.12 | 3.17 | 17.79 | 4.20 |
|  | Anger Expression Index | 23.22 | 9.50 | 26.14 | 10.94 |
| **STAI-Y (Anxiety)** | State Anxiety | 31.17 | 6.17 | 30.68 | 8.49 |
|  | Trait Anxiety | 36.12 | 8.95 | 33.46 | 10.71 |
| **TAS-20 (Alexithymia)** | F1 | 11.63 | 5.42 | 10.57 | 3.41 |
|  | F2 | 11.56 | 5.50 | 11.71 | 4.33 |
|  | F3 | 15.49 | 4.02 | 18.14 | 4.66 |
|  | Total Score | 38.68 | 11.48 | 40.43 | 7.95 |
| **RPM (Fluid intelligence)** | Raw Score | 33.63 | 2.20 | 34.07 | 2.37 |
|  | Corrected Score | 29.81 | 2.45 | 30.17 | 2.19 |
|  | | | | | |
| **Personality tests** | **Subscales** | **mean** | **SD** | **mean** | **SD** |
| **BFQ-2** | Energy | 76.15 | 11.86 | 80.93 | 10.33 |
|  | Agreeableness | 96.68 | 10.04 | 93.29 | 7.94 |
|  | Conscientiousness | 86.90 | 10.99 | 91.89 | 11.70 |
|  | Emotional Stability | 76.44 | 15.55 | 81.68 | 15.80 |
|  | Openness | 88.41 | 13.95 | 92.00 | 12.00 |
|  | Lie | 40.17 | 6.62 | 39.68 | 5.24 |
| **TCI** | NS1 | 6.71 | 2.46 | 6.21 | 1.73 |
|  | NS2 | 4.05 | 2.38 | 3.71 | 2.14 |
|  | NS3 | 5.00 | 1.76 | 4.96 | 1.97 |
|  | NS4 | 3.71 | 1.78 | 4.18 | 1.81 |
|  | NS Total Score | 19.46 | 5.85 | 19.07 | 4.82 |
|  | HA1 | 3.90 | 2.21 | 3.46 | 2.27 |
|  | HA2 | 3.98 | 1.64 | 3.29 | 1.67 |
|  | HA3 | 3.07 | 2.13 | 2.93 | 2.14 |
|  | HA4 | 2.44 | 2.27 | 2.32 | 2.18 |
|  | HA Total Score | 13.39 | 5.50 | 12.00 | 5.92 |
|  | RD1 | 6.68 | 1.81 | 6.32 | 2.18 |
|  | RD3 | 5.51 | 2.11 | 5.46 | 2.32 |
|  | RD4 | 3.22 | 1.19 | 3.75 | 1.17 |
|  | RD Total Score | 15.41 | 3.68 | 15.54 | 4.00 |
|  | PS | 4.95 | 1.47 | 5.57 | 1.53 |
|  | SD1 | 6.56 | 1.58 | 6.29 | 1.86 |
|  | SD2 | 5.93 | 1.97 | 5.68 | 1.81 |
|  | SD3 | 4.56 | 0.92 | 4.36 | 1.22 |
|  | SD4 | 6.83 | 2.83 | 5.68 | 2.93 |
|  | SD5 | 10.07 | 1.81 | 9.64 | 3.16 |
|  | SD Total Score | 33.95 | 6.23 | 31.64 | 8.24 |
|  | C1 | 7.05 | 1.67 | 6.54 | 1.40 |
|  | C2 | 5.54 | 1.12 | 5.43 | 1.45 |
|  | C3 | 6.73 | 1.14 | 6.29 | 1.33 |
|  | C4 | 8.05 | 2.18 | 7.43 | 3.00 |
|  | C5 | 7.80 | 1.19 | 7.11 | 1.26 |
|  | C Total Score | 35.17 | 4.64 | 32.79 | 5.41 |
|  | ST1 | 4.71 | 2.22 | 4.54 | 2.44 |
|  | ST2 | 3.80 | 2.22 | 3.14 | 2.03 |
|  | ST3 | 6.10 | 3.27 | 3.86 | 3.47 |
|  | ST Total Score | 14.61 | 6.58 | 11.54 | 5.87 |

Abbreviations: RS-10, Resilience Scale-10; ASQ, Attachment Style Questionnaire; BDI, Beck’s Depression Inventory; COPE, Coping Orientation to Problems and Experiences; ERQ, Emotion Regulation Questionnaire; GSES, General self-efficacy scale; HAM-A, Hamilton Anxiety Rating Scale; HAM-D, Hamilton Depression Rating Scale; HR-SS, Holmes and Rahe Stress Scale; IRI, Interpersonal Reactivity Index; PANAS, Positive and Negative Affect Schedule; STAXI, State-Trait Anger Expression Inventory; STAI-Y, State-Trait Anxiety Inventory – Form Y; TAS-20, Toronto Alexithymia Scale; RPM, Raven’s progressive matrices. BFQ-2, Big Five Questionnaire-2; TCI, Temperament and Character Inventory; SD: Standard Deviation.

**_______________________________________________________________________________________**


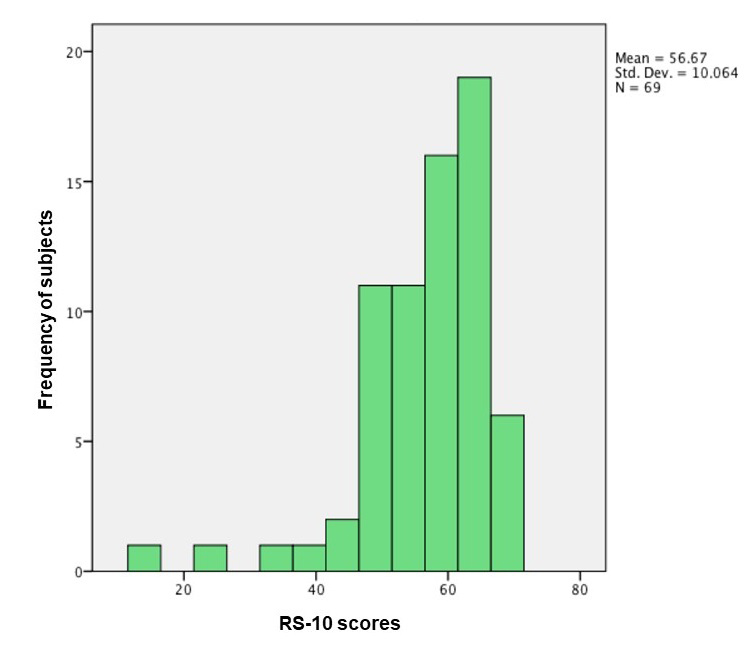


***Figure S1. Bar plot illustrating the frequency distribution of RS-10 scores.***

_______________________________________________________________________________________

| **Table S2. List of neuromorphological variables analyzed in the correlation analyses.** | | |
| --- | --- | --- |
| **Variables** | **Category** | **Sub-category** |
| Left temporal pole | Thickness | Cortical |
| Right middle temporal Cx | Thickness | Cortical |
| Left isthmus cingulate Cx | Thickness | Cortical |
| Right superior temporal Cx | Thickness | Cortical |
| Left entorhinal Cx | Thickness | Cortical |
| Right insula | Thickness | Cortical |
| Left medial orbitofrontal Cx | Thickness | Cortical |
| Left supramarginal gyrus | Thickness | Cortical |
| Right lateral orbitofrontal Cx | Thickness | Cortical |
| Left transverse temporal Cx | Thickness | Cortical |
| Right medial orbitofrontal Cx | Thickness | Cortical |
| Left cuneus | Thickness | Cortical |
| Left precuneus | Thickness | Cortical |
| Left parahippocampal gyrus | Thickness | Cortical |
| Left inferior parietal Cx | Thickness | Cortical |
| Right paracentral Cx | Thickness | Cortical |
| Right pars orbitalis | Thickness | Cortical |
| Left lingual gyrus | Thickness | Cortical |
| Left lateral orbitofrontal Cx | Thickness | Cortical |
| Right entorhinal Cx | Thickness | Cortical |
| Left lateral occipital Cx | Thickness | Cortical |
| Right supramarginal gyrus | Thickness | Cortical |
| Right pericalcarine | Thickness | Cortical |
| Left pars orbitalis | Thickness | Cortical |
| Left superior temporal Cx | Thickness | Cortical |
| Right isthmus cingulate Cx | Thickness | Cortical |
| Left caudal anterior cingulate Cx | Thickness | Cortical |
| Left posterior cingulate Cx | Thickness | Cortical |
| Right parahippocampal gyrus | Thickness | Cortical |
| Right rostral middle frontal Cx | Thickness | Cortical |
| Right postcentral Cx | Thickness | Cortical |
| Right precuneus | Thickness | Cortical |
| Left rostral middle frontal Cx | Thickness | Cortical |
| Left pericalcarine Cx | Thickness | Cortical |
| Right inferior temporal thickness | Thickness | Cortical |
| Left rostral anterior cingulate | Thickness | Cortical |
| Left fusiform gyrus | Thickness | Cortical |
| Left postcentral Cx | Thickness | Cortical |
| Left pars triangularis | Thickness | Cortical |
| Right caudal middle frontal Cx | Thickness | Cortical |
| Left pars opercularis | Thickness | Cortical |
| Right lateral occipital Cx | Thickness | Cortical |
| Right lingual gyrus | Thickness | Cortical |
| Right posterior cingulate Cx | Thickness | Cortical |
| Right rostral anterior cingulate Cx | Thickness | Cortical |
| Right frontal pole | Thickness | Cortical |
| Left superior frontal Cx | Thickness | Cortical |
| Right superior parietal | Thickness | Cortical |
| Right precentral Cx | Thickness | Cortical |
| Right transverse temporal Cx | Thickness | Cortical |
| Left paracentral Cx | Thickness | Cortical |
| Left caudal middle frontal Cx | Thickness | Cortical |
| Left middle temporal gyrus | Thickness | Cortical |
| Left inferior temporal Cx | Thickness | Cortical |
| Right cuneus | Thickness | Cortical |
| Right temporal pole | Thickness | Cortical |
| Right caudal anterior cingulate Cx | Thickness | Cortical |
| Right pars opercularis | Thickness | Cortical |
| Left superior parietal Cx | Thickness | Cortical |
| Right inferior parietal Cx | Thickness | Cortical |
| Left precentral Cx | Thickness | Cortical |
| Right fusiform gyrus | Thickness | Cortical |
| Right pars triangularis | Thickness | Cortical |
| Right superior frontal Cx | Thickness | Cortical |
| Left frontal pole | Thickness | Cortical |
| Left medial orbitofrontal Cx | Volume | Cortical |
| Right pars orbitalis | Volume | Cortical |
| Left pars triangularis | Volume | Cortical |
| Right rostral anterior cingulate Cx | Volume | Cortical |
| Right superior temporal Cx | Volume | Cortical |
| Left transverse temporal gyrus | Volume | Cortical |
| Left temporal pole | Volume | Cortical |
| Right inferior temporal Cx | Volume | Cortical |
| Left posterior cingulate Cx | Volume | Cortical |
| Left insula | Volume | Cortical |
| Left superior temporal Cx | Volume | Cortical |
| Right middle temporal Cx | Volume | Cortical |
| Left cuneus | Volume | Cortical |
| Right supramarginal gyrus | Volume | Cortical |
| Right transverse temporal gyrus | Volume | Cortical |
| Left isthmus cingulate Cx | Volume | Cortical |
| Right superior parietal Cx | Volume | Cortical |
| Right lateral orbitofrontal Cx | Volume | Cortical |
| Left lingual gyrus | Volume | Cortical |
| Right posterior cingulate Cx | Volume | Cortical |
| Left supramarginal gyrus | Volume | Cortical |
| Left middle temporal Cx | Volume | Cortical |
| Right pars triangularis | Volume | Cortical |
| Right caudal anterior cingulate Cx | Volume | Cortical |
| Left superior parietal Cx | Volume | Cortical |
| Right lingual gyrus | Volume | Cortical |
| Left lateral orbitofrontal Cx | Volume | Cortical |
| Right rostral middle frontal Cx | Volume | Cortical |
| Left entorhinal Cx | Volume | Cortical |
| Right superior frontal Cx | Volume | Cortical |
| Right isthmus cingulate Cx | Volume | Cortical |
| Left precentral Cx | Volume | Cortical |
| Right precentral Cx | Volume | Cortical |
| Right entorhinal Cx | Volume | Cortical |
| Left superior frontal Cx | Volume | Cortical |
| Left insula | Volume | Cortical |
| Left rostral middle frontal Cx | Volume | Cortical |
| Left pars opercularis | Volume | Cortical |
| Left inferior temporal Cx | Volume | Cortical |
| Left pericalcarine Cx | Volume | Cortical |
| Left caudal middle frontal Cx | Volume | Cortical |
| Left pars orbitalis | Volume | Cortical |
| Right pericalcarine Cx | Volume | Cortical |
| Left lateral occipital Cx | Volume | Cortical |
| Right fusiform gyrus | Volume | Cortical |
| Left postcentral Cx | Volume | Cortical |
| Right cuneus | Volume | Cortical |
| Right paracentral Cx | Volume | Cortical |
| Right medial orbitofrontal Cx | Volume | Cortical |
| Left fusiform gyrus | Volume | Cortical |
| Left rostral anterior cingulate Cx | Volume | Cortical |
| Right lateral occipital Cx | Volume | Cortical |
| Right precuneus | Volume | Cortical |
| Right parahippocampal gyrus | Volume | Cortical |
| Left paracentral Cx | Volume | Cortical |
| Right pars opercularis | Volume | Cortical |
| Left inferiorparietal Cx | Volume | Cortical |
| Right inferior parietal Cx | Volume | Cortical |
| Right temporal pole | Volume | Cortical |
| Left caudal anterior cingulate Cx | Volume | Cortical |
| Right frontal pole | Volume | Cortical |
| Left precuneus | Volume | Cortical |
| Left frontal pole | Volume | Cortical |
| Right postcentral Cx | Volume | Cortical |
| Right caudal middle frontal Cx | Volume | Cortical |
| Left amygdala | Volume | Subcortical |
| Right hippocampus | Volume | Subcortical |
| Corpus callosum - central | Volume | Subcortical |
| Right insula | Volume | Subcortical |
| Left hippocampus | Volume | Subcortical |
| Corpus callosum - mid-posterior | Volume | Subcortical |
| Corpus callosum - mid-anterior | Volume | Subcortical |
| Left thalamus | Volume | Subcortical |
| Right amygdala | Volume | Subcortical |
| Brainstem | Volume | Subcortical |
| Left cerebellar white matter | Volume | Subcortical |
| Right thalamus | Volume | Subcortical |
| Corpus callosum - Anterior | Volume | Subcortical |
| Corpus callosum - Anterior | Volume | Subcortical |
| Right cerebellar white matter | Volume | Subcortical |
| Right cerebellar cortex | Volume | Subcortical |
| Left ventral diencephalon | Volume | Subcortical |
| Right pallidum | Volume | Subcortical |
| Left caudate | Volume | Subcortical |
| Right putamen | Volume | Subcortical |
| Left putamen | Volume | Subcortical |
| Right caudate | Volume | Subcortical |
| Left cerebellar cortex | Volume | Subcortical |
| Left parahippocampal gyrus | Volume | Subcortical |
| Right accumbens | Volume | Subcortical |
| Left pallidum | Volume | Subcortical |
| Left nucleus accumbens | Volume | Subortical |
| Right cerebellar lobules I-II | Thickness | Cerebellum |
| Right cerebellar lobule X | Thickness | Cerebellum |
| Left cerebellar lobules I-II | Thickness | Cerebellum |
| Left cerebellar lobule V | Thickness | Cerebellum |
| Right cerebellar lobule IV | Thickness | Cerebellum |
| Right cerebellar lobule VI | Thickness | Cerebellum |
| Left cerebellar lobule VI | Thickness | Cerebellum |
| Left cerebellar lobule IX | Thickness | Cerebellum |
| Left cerebellar lobule VIIIb | Thickness | Cerebellum |
| Left cerebellar lobule IV | Thickness | Cerebellum |
| Right cerebellar lobule V | Thickness | Cerebellum |
| Right cerebellar lobule VIIIb | Thickness | Cerebellum |
| Left cerebellar lobule X | Thickness | Cerebellum |
| Right Crus I | Thickness | Cerebellum |
| Right Crus II | Thickness | Cerebellum |
| Right cerebellar lobule III | Thickness | Cerebellum |
| Right cerebellar lobule IX | Thickness | Cerebellum |
| Left Crus II | Thickness | Cerebellum |
| Left cerebellar lobule VIIIa | Thickness | Cerebellum |
| Left cerebellar lobule III | Thickness | Cerebellum |
| Left Crus I | Thickness | Cerebellum |
| Right cerebellar lobule VIIb | Thickness | Cerebellum |
| Right cerebellar lobule VIIIa | Thickness | Cerebellum |
| Left cerebellar lobule VIIb | Thickness | Cerebellum |
| Left cerebellar lobule X | Volume | Cerebellum |
| Right cerebellar lobule X | Volume | Cerebellum |
| Right cerebellar lobule VIIb | Volume | Cerebellum |
| Left Crus II | Volume | Cerebellum |
| Right cerebellar lobule VIIIa | Volume | Cerebellum |
| Left cerebellar lobule VI | Volume | Cerebellum |
| Right Crus I | Volume | Cerebellum |
| Left Crus I | Volume | Cerebellum |
| Right cerebellar lobules I-II | Volume | Cerebellum |
| Right Crus II | Volume | Cerebellum |
| Right cerebellar lobule VI | Volume | Cerebellum |
| Left cerebellar lobule I-II | Volume | Cerebellum |
| Left cerebellar lobule IV | Volume | Cerebellum |
| Left cerebellar lobule VIIb | Volume | Cerebellum |
| Left cerebellar lobule V | Volume | Cerebellum |
| Right cerebellar lobule V | Volume | Cerebellum |
| Right cerebellar lobule VIIIb | Volume | Cerebellum |
| Left cerebellar lobule VIIIa | Volume | Cerebellum |
| Right ventral diencephalon | Volume | Cerebellum |
| Right cerebellar lobule IX | Volume | Cerebellum |
| Left cerebellar lobule VIIIb | Volume | Cerebellum |
| Left cerebellar lobule III | Volume | Cerebellum |
| Right cerebellar lobule IV | Volume | Cerebellum |
| Right cerebellar lobule III | Volume | Cerebellum |
| Left cerebellar lobule IX | Volume | Cerebellum |

Abbreviations: Cx, Cortex.

_______________________________________________________________________________________

| **Table S3. Correlations between the dependent variable RS-10 and the 279 variables of interest** (in **bold** the significant correlations)**.** | | | | | |
| --- | --- | --- | --- | --- | --- |
| *Abbreviations are the same as in Fig.1, Table S1 and S2.* | | | | | |
|  | **Variables** | **Category** | **Spearman's rho** | **p-value** | **FDR adjusted p-value** |
|  | GSES | Psychological dimension | 0.6507 | **<0.00001** | **<0.00001** |
|  | PANAS - Positive Affect | Psychological dimension | 0.6384 | **<0.00001** | **<0.00001** |
|  | STAI-Y - Trait anxiety | Psychological dimension | -0.6050 | **<0.00001** | **<0.00001** |
|  | IRI - Personal Distress | Psychological dimension | -0.5485 | **<0.00001** | **0.0001** |
|  | STAI-Y - State Anxiety | Psychological dimension | -0.5249 | **<0.00001** | **0.0002** |
|  | ASQ - Need for Approval | Psychological dimension | -0.5244 | **<0.00001** | **0.0002** |
|  | ASQ -Confidence | Psychological dimension | 0.4969 | **0.00001** | **0.0006** |
|  | TCI - HA1 | Personality trait | -0.4844 | **0.00002** | **0.0008** |
|  | BFQ-2 Energy | Personality trait | 0.4821 | **0.00003** | **0.0008** |
|  | TCI - HA | Personality trait | -0.4804 | **0.00003** | **0.0008** |
|  | TCI - SD3 | Personality trait | 0.4767 | **0.00003** | **0.0009** |
|  | PANAS - Negative Affect | Psychological dimension | -0.4368 | **0.0002** | **0.0041** |
|  | ASQ - Preoccupation with Relationships | Psychological dimension | -0.4301 | **0.0002** | **0.0048** |
|  | BFQ-2 Emotional Stability | Personality trait | 0.4123 | **0.0004** | **0.0086** |
|  | HAM-D | Psychological dimension | -0.3991 | **0.0007** | **0.0127** |
|  | STAXI - State Anger | Psychological dimension | -0.3952 | **0.0008** | **0.0135** |
|  | ASQ - Relationships as Secondary | Psychological dimension | -0.3756 | **0.0015** | **0.0242** |
|  | COPE - Problem solving | Psychological dimension | 0.3714 | **0.0017** | **0.0260** |
|  | TCI - SD2 | Personality trait | 0.3630 | **0.0022** | **0.0320** |
|  | BFQ-2 Openness | Personality trait | 0.3569 | **0.0026** | **0.0364** |
|  | TCI - HA2 | Personality trait | -0.3546 | **0.0028** | **0.0372** |
|  | Left lobule X | Cerebellar Volume | -0.3513 | **0.0031** | **0.0388** |
|  | BFQ-2 Conscientiousness | Personality trait | 0.3500 | **0.0032** | **0.0388** |
|  | TCI - SD | Personality trait | 0.3467 | **0.0035** | **0.0409** |
|  | Left medial orbitofrontal Cx | Cortical Volume | 0.3425 | **0.0040** | **0.0443** |
|  | COPE Avoidance | Psychological dimension | -0.3358 | **0.0048** | 0.0515 |
|  | BFQ-2 Lie | Personality trait | 0.3311 | **0.0054** | 0.0563 |
|  | Left amygdala | Subcortical Volume | 0.3246 | **0.0065** | 0.0648 |
|  | BFQ-2 Agreeableness | Personality trait | 0.3212 | **0.0071** | 0.0686 |
|  | BDI - Total score | Psychological dimension | -0.3191 | **0.0075** | 0.0700 |
|  | Left temporal pole | Cortical thickness | 0.3155 | **0.0083** | 0.0745 |
|  | Right lobules I-II | Cerebellar Cortical thickness | -0.3037 | **0.0112** | 0.0976 |
|  | Right hippocampus | Subcortical Volume | 0.2964 | **0.0134** | 0.1132 |
|  | TCI - NS3 | Personality trait | 0.2831 | **0.0184** | 0.1510 |
|  | TCI - HA4 | Personality trait | -0.2659 | **0.0272** | 0.2170 |
|  | ERQ Expressive Suppression | Psychological dimension | 0.2606 | **0.0305** | 0.2345 |
|  | Right pars orbitalis | Cortical Volume | 0.2591 | **0.0316** | 0.2345 |
|  | Right lobule X | Cerebellar Cortical thickness | -0.2586 | **0.0319** | 0.2345 |
|  | Corpus callosum - central | Subcortical Volume | 0.2544 | **0.0349** | 0.2497 |
|  | HAM-A | Psychological dimension | -0.2510 | **0.0375** | 0.2523 |
|  | TAS-20 Total score | Psychological dimension | -0.2507 | **0.0377** | 0.2523 |
|  | TCI - SD5 | Personality trait | 0.2490 | **0.0391** | 0.2523 |
|  | Right lobule X | Cerebellar Volume | -0.2484 | **0.0396** | 0.2523 |
|  | Right insula | Cortical Volume | 0.2482 | **0.0398** | 0.2523 |
|  | Left lobules I-II | Cerebellar Cortical thickness | -0.2434 | **0.0439** | 0.2685 |
|  | Right lobule VIIb | Cerebellar Volume | -0.2430 | **0.0443** | 0.2685 |
|  | TCI - PS | Personality trait | 0.2357 | 0.0512 | 0.3041 |
|  | Left hippocampus | Subcortical Volume | 0.2341 | 0.0529 | 0.3073 |
|  | Corpus callosum - mid-posterior | Subcortical Volume | 0.2323 | 0.0548 | 0.3113 |
|  | Left pars triangularis | Cortical Volume | 0.2300 | 0.0572 | 0.3113 |
|  | Right rostral anterior cingulate Cx | Cortical Volume | 0.2296 | 0.0577 | 0.3113 |
|  | Right superior temporal Cx | Cortical Volume | 0.2293 | 0.0580 | 0.3113 |
|  | Left transverse temporal gyrus | Cortical Volume | 0.2281 | 0.0594 | 0.3129 |
|  | Left temporal pole | Cortical Volume | 0.2270 | 0.0607 | 0.3137 |
|  | Left Crus II | Cerebellar Volume | -0.2238 | 0.0646 | 0.3189 |
|  | Right inferior temporal Cx | Cortical Volume | 0.2237 | 0.0646 | 0.3189 |
|  | Left posterior cingulate Cx | Cortical Volume | 0.2233 | 0.0652 | 0.3189 |
|  | Left insula | Cortical Volume | 0.2207 | 0.0684 | 0.3239 |
|  | TAS-20 F2 | Psychological dimension | -0.2206 | 0.0685 | 0.3239 |
|  | TCI - SD1 | Personality trait | 0.2187 | 0.0710 | 0.3261 |
|  | ASQ - Discomfort with Closeness | Psychological dimension | -0.2185 | 0.0713 | 0.3261 |
|  | Corpus callosum - mid-anterior | Subcortical Volume | 0.2168 | 0.0736 | 0.3311 |
|  | COPE - Turning to Religion | Psychological dimension | -0.2128 | 0.0792 | 0.3457 |
|  | Left thalamus | Subcortical Volume | 0.2118 | 0.0806 | 0.3457 |
|  | Left superior temporal Cx | Cortical Volume | 0.2112 | 0.0816 | 0.3457 |
|  | RPM Raw scores | Psychological dimension | 0.2110 | 0.0818 | 0.3457 |
|  | Right middle temporal Cx | Cortical Volume | 0.2052 | 0.0908 | 0.3748 |
|  | Left lobule V | Cerebellar Cortical thickness | -0.2047 | 0.0916 | 0.3748 |
|  | Left cuneus | Cortical Volume | 0.2038 | 0.0931 | 0.3748 |
|  | ERQ - Expressive Suppression | Psychological dimension | -0.2032 | 0.0940 | 0.3748 |
|  | Right supramarginal gyrus | Cortical Volume | 0.1995 | 0.1002 | 0.3938 |
|  | Right transverse temporal gyrus | Cortical Volume | 0.1978 | 0.1033 | 0.4002 |
|  | Right lobule IV | Cerebellar Cortical thickness | -0.1942 | 0.1098 | 0.4195 |
|  | Right middle temporal Cx | Cortical thickness | 0.1910 | 0.1160 | 0.4318 |
|  | Right lobule VIIIa | Cerebellar Cortical Volume | -0.1909 | 0.1161 | 0.4318 |
|  | Left isthmus cingulate Cx | Cortical Volume | 0.1885 | 0.1209 | 0.4420 |
|  | Right superior parietal Cx | Cortical Volume | 0.1877 | 0.1226 | 0.4420 |
|  | Left lobule VI | Cerebellar Cortical Volume | -0.1866 | 0.1246 | 0.4420 |
|  | Right Crus I | Cerebellar Volume | -0.1861 | 0.1257 | 0.4420 |
|  | Right lobule VI | Cerebellar Cortical thickness | -0.1856 | 0.1267 | 0.4420 |
|  | Left cerebellar lobule VI | Cortical thickness | -0.1839 | 0.1305 | 0.4494 |
|  | TCI - C | Personality trait | 0.1802 | 0.1383 | 0.4637 |
|  | TCI - C4 | Personality trait | 0.1797 | 0.1395 | 0.4637 |
|  | Right amygdala | Subcortical Volume | 0.1785 | 0.1422 | 0.4637 |
|  | Left Crus I | Cerebellar Volume | -0.1781 | 0.1431 | 0.4637 |
|  | Left lobule IX | Cerebellar Cortical thickness | -0.1780 | 0.1434 | 0.4637 |
|  | Right lateral orbitofrontal Cx | Cortical Volume | 0.1775 | 0.1446 | 0.4637 |
|  | TAS-20 F3 | Psychological dimension | -0.1764 | 0.1471 | 0.4665 |
|  | Right lobules I-II | Cerebellar Volume | -0.1737 | 0.1534 | 0.4810 |
|  | COPE - Positive Attitude | Psychological dimension | 0.1708 | 0.1606 | 0.4932 |
|  | Left lingual gyrus | Cortical Volume | 0.1700 | 0.1624 | 0.4932 |
|  | Right posterior cingulate Cx | Cortical Volume | 0.1678 | 0.1682 | 0.4932 |
|  | Right Crus II | Cerebellar Volume | -0.1671 | 0.1698 | 0.4932 |
|  | TCI - C3 | Personality trait | 0.1668 | 0.1708 | 0.4932 |
|  | Left supramarginal gyrus | Cortical Volume | 0.1664 | 0.1718 | 0.4932 |
|  | Brainstem | Subcortical Volume | 0.1661 | 0.1726 | 0.4932 |
|  | RPM Corrected scores | Psychological dimension | 0.1661 | 0.1726 | 0.4932 |
|  | Left middle temporal Cx | Cortical Volume | 0.1658 | 0.1732 | 0.4932 |
|  | Right lobule VI | Cerebellar Volume | -0.1635 | 0.1795 | 0.5012 |
|  | Left white matter | Cerebellar Volume | 0.1634 | 0.1796 | 0.5012 |
|  | Right pars triangularis | Cortical Volume | 0.1614 | 0.1852 | 0.5117 |
|  | Right caudal anterior cingulate Cx | Cortical Volume | 0.1594 | 0.1909 | 0.5222 |
|  | Left superior parietal Cx | Cortical Volume | 0.1551 | 0.2031 | 0.5460 |
|  | Right lingual gyrus | Cortical Volume | 0.1550 | 0.2035 | 0.5460 |
|  | Left lateral orbitofrontal Cx | Cortical Volume | 0.1504 | 0.2174 | 0.5742 |
|  | Right rostral middle frontal Cx | Cortical Volume | 0.1502 | 0.2181 | 0.5742 |
|  | Left entorhinal Cx | Cortical Volume | 0.1481 | 0.2246 | 0.5746 |
|  | Right superior frontal Cx | Cortical Volume | 0.1478 | 0.2255 | 0.5746 |
|  | Right thalamus | Subcortical Volume | 0.1472 | 0.2274 | 0.5746 |
|  | Right isthmus cingulate Cx | Cortical Volume | 0.1462 | 0.2305 | 0.5746 |
|  | Left nucleus accumbens | Subcortical Volume | -0.1462 | 0.2308 | 0.5746 |
|  | Left isthmus cingulate Cx | Cortical thickness | 0.1460 | 0.2312 | 0.5746 |
|  | Left precentral Cx | Cortical Volume | 0.1450 | 0.2344 | 0.5746 |
|  | Left lobule I-II | Cerebellar Volume | -0.1440 | 0.2378 | 0.5746 |
|  | Right precentral Cx | Cortical Volume | 0.1438 | 0.2385 | 0.5746 |
|  | Right superior temporal Cx | Cortical thickness | 0.1437 | 0.2389 | 0.5746 |
|  | TAS-20 F1 | Psychological dimension | -0.1412 | 0.2471 | 0.5770 |
|  | Left entorhinal Cx | Cortical thickness | 0.1408 | 0.2486 | 0.5770 |
|  | Right insula | Cortical thickness | 0.1400 | 0.2512 | 0.5770 |
|  | Corpus callosum - Anterior | Subcortical Volume | 0.1400 | 0.2514 | 0.5770 |
|  | Right entorhinal Cx | Cortical Volume | 0.1395 | 0.2530 | 0.5770 |
|  | Left superior frontal Cx | Cortical Volume | 0.1393 | 0.2535 | 0.5770 |
|  | Left lobule IV | Cerebellar Volume | 0.1386 | 0.2559 | 0.5770 |
|  | Left lobule VIIb | Cerebellar Volume | -0.1385 | 0.2564 | 0.5770 |
|  | Left insula | Cortical Volume | 0.1374 | 0.2604 | 0.5789 |
|  | Left lobule VIIIb | Cerebellar Cortical thickness | -0.1367 | 0.2628 | 0.5789 |
|  | Left rostral middle frontal Cx | Cortical Volume | 0.1363 | 0.2642 | 0.5789 |
|  | Left medial orbitofrontal Cx | Cortical thickness | 0.1354 | 0.2674 | 0.5789 |
|  | Left lobule IV | Cerebellar Cortical thickness | -0.1353 | 0.2676 | 0.5789 |
|  | Left pars opercularis | Cortical Volume | 0.1339 | 0.2727 | 0.5852 |
|  | Left inferior temporal Cx | Cortical Volume | 0.1332 | 0.2754 | 0.5864 |
|  | COPE - Social Support | Psychological dimension | -0.1313 | 0.2822 | 0.5933 |
|  | Right lobule V | Cerebellar Cortical thickness | -0.1311 | 0.2828 | 0.5933 |
|  | Left pericalcarine Cx | Cortical Volume | 0.1292 | 0.2902 | 0.5992 |
|  | Left caudal middle frontal Cx | Cortical Volume | 0.1283 | 0.2935 | 0.5992 |
|  | TCI - SD4 | Personality trait | 0.1273 | 0.2971 | 0.5992 |
|  | TCI - HA3 | Personality trait | -0.1273 | 0.2971 | 0.5992 |
|  | Corpus callosum - Anterior | Subcortical Volume | 0.1270 | 0.2985 | 0.5992 |
|  | STAXI - Trait Anger | Psychological dimension | -0.1252 | 0.3053 | 0.5992 |
|  | TCI - NS | Personality trait | 0.1245 | 0.3080 | 0.5992 |
|  | Left pars orbitalis | Cortical Volume | 0.1245 | 0.3082 | 0.5992 |
|  | Right pericalcarine Cx | Cortical Volume | 0.1244 | 0.3086 | 0.5992 |
|  | Left lateral occipital Cx | Cortical Volume | 0.1244 | 0.3087 | 0.5992 |
|  | Right lobule VIIIb | Cerebellar Cortical thickness | -0.1240 | 0.3102 | 0.5992 |
|  | Left lobule V | Cerebellar Volume | -0.1236 | 0.3114 | 0.5992 |
|  | Left lobule X | Cerebellar Cortical thickness | -0.1219 | 0.3186 | 0.6087 |
|  | IRI - Fantasy | Psychological dimension | -0.1208 | 0.3229 | 0.6117 |
|  | Left supramarginal gyrus | Cortical thickness | -0.1204 | 0.3245 | 0.6117 |
|  | Right lateral orbitofrontal Cx | Cortical thickness | 0.1183 | 0.3330 | 0.6218 |
|  | Left transverse temporal Cx | Cortical thickness | 0.1180 | 0.3343 | 0.6218 |
|  | Right medial orbitofrontal Cx | Cortical thickness | 0.1159 | 0.3430 | 0.6338 |
|  | Left cuneus | Cortical thickness | 0.1140 | 0.3508 | 0.6438 |
|  | Right fusiform gyrus | Cortical Volume | 0.1133 | 0.3541 | 0.6458 |
|  | Left postcentral Cx | Cortical Volume | 0.1120 | 0.3595 | 0.6491 |
|  | TCI - ST2 | Personality trait | 0.1117 | 0.3606 | 0.6491 |
|  | Right cuneus | Cortical Volume | 0.1109 | 0.3644 | 0.6499 |
|  | Right paracentral Cx | Cortical Volume | 0.1106 | 0.3657 | 0.6499 |
|  | Right medial orbitofrontal Cx | Cortical Volume | 0.1076 | 0.3787 | 0.6688 |
|  | Left precuneus | Cortical thickness | -0.1042 | 0.3942 | 0.6917 |
|  | TCI - RD3 | Personality trait | 0.1011 | 0.4083 | 0.7076 |
|  | Left fusiform gyrus | Cortical Volume | 0.1005 | 0.4112 | 0.7076 |
|  | TCI - RD1 | Personality trait | -0.1005 | 0.4115 | 0.7076 |
|  | Right white matter | Cerebellar Volume | 0.1000 | 0.4134 | 0.7076 |
|  | Left parahippocampal gyrus | Cortical thickness | -0.0985 | 0.4208 | 0.7159 |
|  | Left rostral anterior cingulate Cx | Cortical Volume | 0.0976 | 0.4252 | 0.7189 |
|  | HR-SS | Psychological dimension | -0.0960 | 0.4324 | 0.7268 |
|  | Right lateral occipital Cx | Cortical Volume | 0.0930 | 0.4473 | 0.7473 |
|  | Right lobule V | Cerebellar Volume | -0.0911 | 0.4564 | 0.7579 |
|  | Right lobule VIIIb | Cerebellar Volume | -0.0890 | 0.4671 | 0.7679 |
|  | Right precuneus | Cortical Volume | 0.0888 | 0.4679 | 0.7679 |
|  | IRI - Perspective Taking | Psychological dimension | 0.0853 | 0.4858 | 0.7872 |
|  | Left inferior parietal Cx | Cortical thickness | -0.0848 | 0.4884 | 0.7872 |
|  | Right parahippocampal gyrus | Cortical Volume | 0.0848 | 0.4885 | 0.7872 |
|  | TCI - NS1 | Personality trait | 0.0838 | 0.4937 | 0.7872 |
|  | TCI - C1 | Personality trait | 0.0828 | 0.4988 | 0.7872 |
|  | Right paracentral Cx | Cortical thickness | -0.0817 | 0.5045 | 0.7872 |
|  | Right cortex | Cerebellar Volume | 0.0817 | 0.5047 | 0.7872 |
|  | Right pars orbitalis | Cortical thickness | 0.0813 | 0.5065 | 0.7872 |
|  | Left ventral diencephalon | Subcortical Volume | 0.0813 | 0.5066 | 0.7872 |
|  | Right Crus I | Cerebellar Cortical thickness | 0.0811 | 0.5079 | 0.7872 |
|  | Left lingual gyrus | Cortical thickness | 0.0777 | 0.5256 | 0.8085 |
|  | Left paracentral Cx | Cortical Volume | 0.0774 | 0.5274 | 0.8085 |
|  | Right Crus II | Cerebellar Cortical thickness | 0.0763 | 0.5331 | 0.8127 |
|  | Right pars opercularis | Cortical Volume | 0.0731 | 0.5506 | 0.8285 |
|  | Left lateral orbitofrontal Cx | Cortical thickness | 0.0729 | 0.5519 | 0.8285 |
|  | Right entorhinal Cx | Cortical thickness | 0.0728 | 0.5523 | 0.8285 |
|  | Left lateral occipital Cx | Cortical thickness | 0.0716 | 0.5586 | 0.8334 |
|  | Right supramarginal gyrus | Cortical thickness | -0.0711 | 0.5616 | 0.8334 |
|  | Right pericalcarine | Cortical thickness | 0.0699 | 0.5682 | 0.8336 |
|  | STAXI - Anger Expression Index | Psychological dimension | -0.0695 | 0.5704 | 0.8336 |
|  | TCI - C2 | Personality trait | 0.0694 | 0.5707 | 0.8336 |
|  | Left lobule VIIIa | Cerebellar Volume | -0.0680 | 0.5789 | 0.8381 |
|  | Right lobule III | Cerebellar Cortical thickness | -0.0678 | 0.5798 | 0.8381 |
|  | Right ventral diencephalon | Subcortical Volume | 0.0657 | 0.5916 | 0.8508 |
|  | TCI - C5 | Personality trait | -0.0636 | 0.6039 | 0.8641 |
|  | Right lobule IX | Cerebellar Cortical thickness | -0.0596 | 0.6268 | 0.8900 |
|  | IRI - Empathic Concern | Psychological dimension | 0.0591 | 0.6293 | 0.8900 |
|  | Left pars orbitalis | Cortical thickness | 0.0584 | 0.6337 | 0.8900 |
|  | Left superior temporal Cx | Cortical thickness | 0.0581 | 0.6355 | 0.8900 |
|  | Right isthmus cingulate Cx | Cortical thickness | 0.0576 | 0.6380 | 0.8900 |
|  | Left caudal anterior cingulate Cx | Cortical thickness | -0.0556 | 0.6501 | 0.8985 |
|  | Right pallidum | Subcortical Volume | -0.0555 | 0.6505 | 0.8985 |
|  | Left posterior cingulate Cx | Cortical thickness | -0.0545 | 0.6563 | 0.9020 |
|  | Age | Socio-demographic variable | -0.0540 | 0.6596 | 0.9021 |
|  | Left caudate | Subcortical Volume | 0.0527 | 0.6670 | 0.9077 |
|  | Right putamen | Subcortical Volume | 0.0520 | 0.6714 | 0.9090 |
|  | Right parahippocampal gyrus | Cortical thickness | -0.0515 | 0.6744 | 0.9090 |
|  | TCI - ST3 | Personality trait | -0.0508 | 0.6782 | 0.9091 |
|  | Right rostral middle frontal Cx | Cortical thickness | 0.0496 | 0.6854 | 0.9091 |
|  | Left inferiorparietal Cx | Cortical Volume | -0.0495 | 0.6863 | 0.9091 |
|  | Right inferior parietal Cx | Cortical Volume | 0.0489 | 0.6898 | 0.9091 |
|  | Right temporal pole | Cortical Volume | 0.0483 | 0.6932 | 0.9091 |
|  | Right postcentral Cx | Cortical thickness | -0.0482 | 0.6940 | 0.9091 |
|  | Left Crus II | Cerebellar Cortical thickness | 0.0469 | 0.7022 | 0.9140 |
|  | Right precuneus | Cortical thickness | -0.0460 | 0.7073 | 0.9140 |
|  | Left rostral middle frontal Cx | Cortical thickness | 0.0460 | 0.7076 | 0.9140 |
|  | Left lobule VIIIa | Cerebellar Cortical thickness | -0.0453 | 0.7115 | 0.9141 |
|  | Left putamen | Subcortical Volume | 0.0439 | 0.7204 | 0.9141 |
|  | Left lobule III | Cerebellar Cortical thickness | -0.0433 | 0.7236 | 0.9141 |
|  | Left caudal anterior cingulate Cx | Cortical Volume | 0.0433 | 0.7239 | 0.9141 |
|  | Left pericalcarine Cx | Cortical thickness | 0.0433 | 0.7241 | 0.9141 |
|  | Right inferior temporal thickness | Cortical thickness | 0.0417 | 0.7335 | 0.9208 |
|  | Left rostral anterior cingulate | Cortical thickness | 0.0408 | 0.7391 | 0.9208 |
|  | Left fusiform gyrus | Cortical thickness | 0.0408 | 0.7394 | 0.9208 |
|  | TCI - NS4 | Personality trait | -0.0403 | 0.7426 | 0.9208 |
|  | Right lobule IX | Cerebellar Volume | -0.0393 | 0.7487 | 0.9243 |
|  | Left Crus I | Cerebellar Cortical thickness | 0.0382 | 0.7552 | 0.9282 |
|  | Left postcentral Cx | Cortical thickness | 0.0371 | 0.7625 | 0.9304 |
|  | Right caudate | Subcortical Volume | 0.0366 | 0.7651 | 0.9304 |
|  | Left pars triangularis | Cortical thickness | -0.0363 | 0.7670 | 0.9304 |
|  | Right caudal middle frontal Cx | Cortical thickness | 0.0357 | 0.7709 | 0.9311 |
|  | Left lobule VIIIb | Cerebellar Volume | -0.0320 | 0.7941 | 0.9470 |
|  | TCI - NS2 | Personality trait | 0.0316 | 0.7963 | 0.9470 |
|  | Right frontal pole | Cortical Volume | -0.0315 | 0.7970 | 0.9470 |
|  | Left pars opercularis | Cortical thickness | 0.0314 | 0.7977 | 0.9470 |
|  | Left lobule III | Cerebellar Volume | -0.0302 | 0.8052 | 0.9490 |
|  | Right lateral occipital Cx | Cortical thickness | -0.0297 | 0.8089 | 0.9490 |
|  | Right lingual gyrus | Cortical thickness | 0.0289 | 0.8134 | 0.9490 |
|  | Right posterior cingulate Cx | Cortical thickness | 0.0288 | 0.8142 | 0.9490 |
|  | Right rostral anterior cingulate Cx | Cortical thickness | 0.0285 | 0.8164 | 0.9490 |
|  | Left cortex | Cerebellar Volume | 0.0267 | 0.8276 | 0.9581 |
|  | Right frontal pole | Cortical thickness | -0.0254 | 0.8359 | 0.9637 |
|  | Left superior frontal Cx | Cortical thickness | 0.0244 | 0.8422 | 0.9644 |
|  | Right superior parietal | Cortical thickness | 0.0239 | 0.8456 | 0.9644 |
|  | Right precentral Cx | Cortical thickness | 0.0237 | 0.8469 | 0.9644 |
|  | TCI - RD4 | Personality trait | 0.0231 | 0.8503 | 0.9644 |
|  | Right transverse temporal Cx | Cortical thickness | 0.0189 | 0.8775 | 0.9819 |
|  | Left precuneus | Cortical Volume | 0.0183 | 0.8814 | 0.9819 |
|  | Left paracentral Cx | Cortical thickness | 0.0180 | 0.8832 | 0.9819 |
|  | Left frontal pole | Cortical Volume | 0.0173 | 0.8875 | 0.9819 |
|  | Right lobule VIIb | Cerebellar Cortical thickness | 0.0165 | 0.8927 | 0.9819 |
|  | Left caudal middle frontal Cx | Cortical thickness | 0.0163 | 0.8943 | 0.9819 |
|  | Right postcentral Cx | Cortical Volume | 0.0161 | 0.8954 | 0.9819 |
|  | Left middle temporal gyrus | Cortical thickness | -0.0158 | 0.8977 | 0.9819 |
|  | Left inferior temporal Cx | Cortical thickness | 0.0148 | 0.9040 | 0.9819 |
|  | Right cuneus | Cortical thickness | -0.0143 | 0.9072 | 0.9819 |
|  | TCI - RD | Personality trait | 0.0142 | 0.9076 | 0.9819 |
|  | Right temporal pole | Cortical thickness | 0.0142 | 0.9080 | 0.9819 |
|  | TCI - ST | Personality trait | 0.0132 | 0.9144 | 0.9849 |
|  | Right caudal anterior cingulate Cx | Cortical thickness | -0.0115 | 0.9251 | 0.9849 |
|  | Right lobule VIIIa | Cerebellar Cortical thickness | -0.0114 | 0.9256 | 0.9849 |
|  | Right pars opercularis | Cortical thickness | 0.0111 | 0.9279 | 0.9849 |
|  | Left lobule VIIb | Cerebellar Cortical thickness | 0.0093 | 0.9397 | 0.9849 |
|  | Left superior parietal Cx | Cortical thickness | -0.0090 | 0.9416 | 0.9849 |
|  | Right lobule IV | Cerebellar Volume | 0.0084 | 0.9456 | 0.9849 |
|  | Right inferior parietal Cx | Cortical thickness | -0.0083 | 0.9462 | 0.9849 |
|  | TCI - ST1 | Personality trait | -0.0077 | 0.9499 | 0.9849 |
|  | Left precentral Cx | Cortical thickness | 0.0068 | 0.9558 | 0.9849 |
|  | Right fusiform gyrus | Cortical thickness | -0.0067 | 0.9561 | 0.9849 |
|  | Right caudal middle frontal Cx | Cortical Volume | -0.0066 | 0.9569 | 0.9849 |
|  | Left parahippocampal gyrus | Subcortical Volume | -0.0063 | 0.9591 | 0.9849 |
|  | Right accumbens | Subcortical Volume | 0.0061 | 0.9602 | 0.9849 |
|  | Right pars triangularis | Cortical thickness | 0.0048 | 0.9687 | 0.9885 |
|  | Left pallidum | Subcortical Volume | -0.0045 | 0.9708 | 0.9885 |
|  | Right superior frontal Cx | Cortical thickness | -0.0034 | 0.9776 | 0.9918 |
|  | Educational Level | Socio-demographic variable | -0.0027 | 0.9826 | 0.9930 |
|  | Left frontal pole | Cortical thickness | 0.0022 | 0.9859 | 0.9930 |
|  | Right lobule III | Cerebellar Volume | 0.0011 | 0.9927 | 0.9962 |
|  | Left lobule IX | Cerebellar Volume | -0.0004 | 0.9976 | 0.9976 |
